# Higher‐order functional connectivity analysis of resting‐state functional magnetic resonance imaging data using multivariate cumulants

**DOI:** 10.1002/hbm.26663

**Published:** 2024-03-23

**Authors:** Rikkert Hindriks, Tommy A. A. Broeders, Menno M. Schoonheim, Linda Douw, Fernando Santos, Wessel van Wieringen, Prejaas K. B. Tewarie

**Affiliations:** ^1^ Department of Mathematics, Faculty of Science Vrije Universiteit Amsterdam Amsterdam The Netherlands; ^2^ Department of Anatomy and Neurosciences, Amsterdam Neuroscience Amsterdam UMC, Vrije Universiteit Amsterdam Amsterdam The Netherlands; ^3^ Dutch Institute for Emergent Phenomena (DIEP) Institute for Advanced Studies, University of Amsterdam Amsterdam The Netherlands; ^4^ Korteweg de Vries Institute for Mathematics University of Amsterdam Amsterdam the Netherlands; ^5^ Department of Epidemiology and Biostatistics Amsterdam UMC, Vrije Universiteit Amsterdam Amsterdam The Netherlands; ^6^ Sir Peter Mansfield Imaging Center School of Physics, University of Nottingham Nottingham United Kingdom; ^7^ Clinical Neurophysiology Group University of Twente Enschede The Netherlands

**Keywords:** functional connectivity, functional MRI, higher‐order connectivity, resting‐state

## Abstract

Blood‐level oxygenation‐dependent (BOLD) functional magnetic resonance imaging (fMRI) is the most common modality to study functional connectivity in the human brain. Most research to date has focused on connectivity between pairs of brain regions. However, attention has recently turned towards connectivity involving more than two regions, that is, higher‐order connectivity. It is not yet clear how higher‐order connectivity can best be quantified. The measures that are currently in use cannot distinguish between pairwise (i.e., second‐order) and higher‐order connectivity. We show that genuine higher‐order connectivity can be quantified by using multivariate cumulants. We explore the use of multivariate cumulants for quantifying higher‐order connectivity and the performance of block bootstrapping for statistical inference. In particular, we formulate a generative model for fMRI signals exhibiting higher‐order connectivity and use it to assess bias, standard errors, and detection probabilities. Application to resting‐state fMRI data from the Human Connectome Project demonstrates that spontaneous fMRI signals are organized into higher‐order networks that are distinct from second‐order resting‐state networks. Application to a clinical cohort of patients with multiple sclerosis further demonstrates that cumulants can be used to classify disease groups and explain behavioral variability. Hence, we present a novel framework to reliably estimate genuine higher‐order connectivity in fMRI data which can be used for constructing hyperedges, and finally, which can readily be applied to fMRI data from populations with neuropsychiatric disease or cognitive neuroscientific experiments.

## Graphical abstract

We introduce and assess the performance of higher‐order functional connectivity metrics using multivariate cumulants for resting‐state functional magnetic resonance imaging (fMRI) data. These metrics quantify connectivity that cannot be explained by pairwise Pearson correlations. Using a generative model we assess the bias, standard errors and detection probabilities in individual and group level data. Application to resting‐state fMRI data demonstrates that spontaneous fMRI signals are organized into higher‐order networks that are distinct from the conventional resting‐state networks. Furthermore, higher‐order connectivity captured relevant non‐redundant information in a clinical cohort of patients with multiple sclerosis.

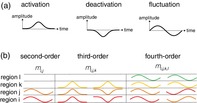

## 1 INTRODUCTION

The term *functional connectivity* refers to the statistical relationship between signals measured at different locations within the brain (Friston, 1994). Functional connectivity in resting‐state fMRI data is usually quantified by the Pearson correlation and has provided many insights about the large‐scale neuronal processes underlying cognition and its disturbances in clinical conditions (Bullmore, 2012; Fox & Greicius, 2010; Smith et al., 2013; van den Heuvel & Hulshoff Pol, 2010). However, studies on functional connectivity of resting‐state fMRI data have almost exclusively focused on connectivity between pairs of brain regions (i.e., second‐order functional connectivity). In particular, network analyses, such as those based on graph‐theory, are only able to capture second‐order connectivity. Many complex systems, however, exhibit higher‐order connectivity, that is, connectivity between more than two regions, and this can lead to new kinds of collective behavior such as explosive phase‐transitions (Battiston et al., 2020; Battiston et al., 2021). It is therefore of interest to know if higher‐order connectivity is present in resting‐state fMRI data, how it is best quantified, and if it can explain behavioral variability in addition to second‐order connectivity. The current study focuses on these questions.

A number of recent studies have investigated higher‐order connectivity in the human brain using resting‐state fMRI. The most commonly used measure for higher‐order connectivity is the *edge connectivity* (Faskowitz et al., 2020), which is defined as the Pearson correlation between two *edge time‐series*. Edge time‐series correspond to unordered pairs of brain regions and are calculated by taking the instantaneous product of the fMRI signals of these regions. Thus, edge connectivity measures the correlation between two *pairs* of brain regions. It has been applied to study higher‐order connectivity between cortical and sub‐cortical structures (Chumin et al., 2022; Korponay & Ph, 2022) and in several clinical populations, including autism spectrum disorder (Esfahlani et al., 2022; Li et al., 2022), auditory processing disorder (Alvand et al., 2022), and stroke patients (Idesis et al., 2022). A related, but more general measure for higher‐order connectivity, was introduced in (Santoro et al., 2022) and quantifies the strength of the connectivity between k brain regions (i.e., k‐th order connectivity) for *k* < 2. Another way of measuring higher‐order connectivity is based on multivariate information theory, which deals with the quantification and analysis of the relationships between multiple variables (Timme et al., 2014) and has been used, for instance, to study ageing (Gatica et al., 2021) and neurodegeneration (Herzog et al., 2022). From an information‐theoretic perspective, higher‐order connectivity is related to the concept of synergy and contrasts the idea that brain regions independently contribute to overall function. Studying synergy in fMRI may provide valuable insights into how different brain regions interact and work together to support various cognitive and complex functions (Herzog et al., 2022; Luppi, Mediano, Rosas, Holland, Fryer, Brien, et al., 2022).

A drawback of the above mentioned measures of higher‐order connectivity is that they do not provide information about whether observed higher‐order connectivity can be reduced to pairwise connectivity of the participating brain regions. For example, for Gaussian fMRI signals, the edge connectivity (Faskowitz et al., 2020) can be expressed in terms of the pairwise correlations between the four participating regions, and as such, is not genuinely of higher‐order. This has been pointed out recently, as Novelli and Razi (Novelli & Razi, 2022) compared edge connectivity networks of resting‐state fMRI signals considered in (Faskowitz et al., 2020) with those obtained under the Gaussian assumption and found that the networks were highly similar. This implies that the observed edge connectivity networks in (Faskowitz et al., 2020) can largely, if not entirely, be explained by pairwise correlations.

More generally, for Gaussian signals, *any* higher‐order connectivity measure can be expressed in terms of pairwise correlations. Since fMRI signals are approximately Gaussian (Hlinka et al., 2011), this raises the question if higher‐order connectivity measures provide any information that is not already contained in the pairwise correlations. Addressing this question requires the use of higher‐order connectivity measures that vanish for Gaussian signals. We will refer to measures with this property as *non‐redundant*. In (Novelli & Razi, 2022) it is shown that a non‐redundant measure of edge connectivity can be obtained by subtracting the Gaussian part from the edge connectivity. A less ad hoc way of measuring non‐redundant higher‐order connectivity is by using multivariate cumulants. Multivariate cumulants characterize the higher‐order correlation structure in multivariate data in a non‐redundant way by subtracting the Gaussian parts of higher‐order multivariate moments. They have been used extensively for analyzing higher‐order correlations in neural spiking activity (Martignon et al., 2000; Yu et al., 2011). In the current study, we explore the use of multivariate cumulants to detect and characterize non‐redundant higher‐order connectivity in resting‐state fMRI data.

Although non‐redundant higher‐order connectivity measures vanish for Gaussian data, their sampling variability requires the use of statistical methods to confirm the presence of higher‐order connectivity in practical applications. For example, although edge connectivity networks and their Gaussian parts were observed to be highly similar (Novelli & Razi, 2022), to conclude that edge networks are in fact redundant requires proper statistical testing. Although this issue has been acknowledged in the neuroimaging community (Jo et al., 2021), there is currently no consensus on how statistical inference is best carried out. The main challenge is that the sampling and null distributions of higher‐order connectivity measures are generally unknown (due to the presence of auto‐correlations in fMRI signals) and cannot be approximated by independent bootstrapping methods (Efron & Tibshirani, 1986). This is particularly problematic when analyzing fMRI data from a single condition (e.g., resting‐state) because the absence of a contrasting condition prevents the use of permutation techniques. In previous studies, different types of randomized data were used and this has led to some discussion about which type of randomization is most appropriate (Betzel et al., 2022; Jo et al., 2021).

We clarify this issue by making explicit the null hypotheses that correspond to different types of randomized data. In the current study, we explore the use of block bootstrapping (Kreiss & Paparoditis, 2011; Kunsch, 1989) for assessing higher‐order connectivity in resting‐state fMRI data and compare it to null‐data obtained using coherent phase‐randomization (Prichard & Theiler, 1994). The advantage of bootstrapping techniques over null‐data is that bootstrapping allows to quantify the level of statistical uncertainty in the connectivity estimates, for example by constructing confidence intervals. Furthermore, as discussed in Section 2.2.2 in more detail, it is challenging to construct null‐data that allows testing the intended null‐hypothesis. In contrast, the sampling distributions obtained from bootstrapping can directly be used to test the null‐hypothesis of no (higher‐order) connectivity.

For practical applications of higher‐order connectivity measures, it is useful to know how these measures, as well as the used statistical inference methods, perform with respect to experimental variables such as scanning duration and physiological properties such as true connectivity strengths. These questions can be addressed by using synthetic multivariate fMRI signals. Such signals need to possess the following three properties. First, since genuine higher‐order connectivity can only be present in non‐Gaussian signals, the synthetic signals need to be non‐Gaussian. Second, the connectivity measure that is of interest can be expressed in terms of the parameters of the generative model. If such an expression is not available, the ground truth with which to compare the analysis results is unknown. Third, the synthetic signals need to have auto‐correlation functions that resemble those of fMRI signals. Without this last property, no conclusions can be drawn about the dependence of performance on scanning duration. In the current study, we use a non‐Gaussian multivariate auto‐regressive process to generate synthetic fMRI signals. This is an ordinary multivariate auto‐regressive process that is driven both by Gaussian and non‐Gaussian noise. The auto‐regressive term introduces auto‐correlations in the synthetic signals, whereas the non‐Gaussian noise introduces higher‐order connectivity. We derive closed‐form expressions for multivariate cumulants and edge connectivity and use them to assess the performance of these measures and the randomization methods.

We apply the methodology to resting‐state BOLD‐fMRI data from the Human Connectome Project (HCP) (Glasser et al., 2013; Glasser, Smith, et al., 2016) and to a clinical cohort of patients suffering from multiple sclerosis (MS). MS is a neuroinflammatory and neurodegenerative disorder of the central nervous system that leads to physical disability and cognitive deterioration strongly affecting day‐to‐day life (Grzegorski & Losy, 2017). Even though white matter lesions are diagnostic hallmarks of MS, their correlation with cognitive impairment is not clear‐cut (Barkhof, 2002). Disease‐related damage to structural pathways has been postulated to induce functional reorganization, particularly involving connections between the default‐mode network (DMN) and the rest of the brain (i.e., extra‐DMN connectivity) (Meijer et al., 2017; Schoonheim et al., 2022). Interestingly, communication between brain regions that are not directly anatomically connected might be more adequately captured using measures that capture how information from multiple brain regions is integrated (Luppi, Mediano, Rosas, Holland, Fryer, Brien, et al., 2022). This is why people with MS may provide a good showcase of the potential clinical relevance of higher‐order connectivity in a clinical sample. Our hypothesis is that higher‐order extra‐DMN connectivity is increased in people with MS who show cognitive impairment.

## 2 METHODS

### 2.1 Theory

#### 2.1.1 Multivariate moments of fMRI signals

In this section, we discuss multivariate moments and interpret them in the context of fMRI signal analysis. With every set of k≥2 fMRI signals, we associate a real number referred to as the k *‐th order moment* of the set of signals, which quantifies the extent to which the signals co‐fluctuate. The second‐order moment of two signals is equal to the covariance between the signals and hence the k‐th order moment can be viewed as a generalization of the covariance to k signals. Moments of order larger than two are referred to as *higher‐order*. By appropriate normalization, the k‐order moment becomes the k *‐th order correlation*. It generalizes the correlation from two to k signals. However, in Section 2.1.3 we discuss that although the k‐order correlation might be an interesting measure for fMRI signal analysis, it does not necessarily vanish if one of the k signals is statistically independent of the other k−1 signals and, in this sense, is redundant. This redundancy can be removed by working with multivariate cumulants instead of with multivariate moments. Multivariate cumulants are discussed in Section 2.1.2. Because multivariate cumulants can be expressed in terms of multivariate moments, the current section serves to familiarize the reader with multivariate moments.

**FIGURE 1:**
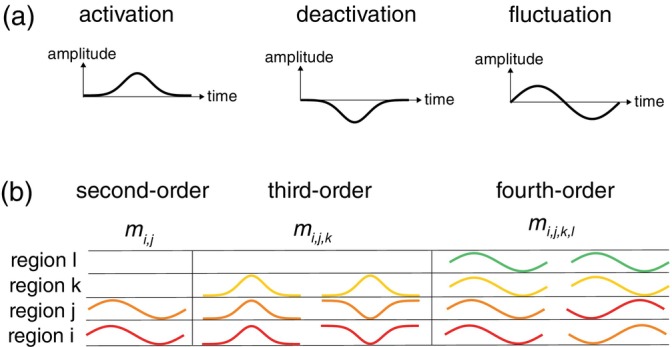
Functional interpretation of multivariate moments. (a) Left: A positive deflection of the functional magnetic resonance imaging (fMRI) signal relative to baseline (activation). Middle: A negative deflection of the fMRI signal relative to baseline (deactivation). Right: The concatenation of an activation and a deactivation (i.e., a fluctuation). (b) Schematic illustration of the functional interpretation of higher‐order moments. A positive second‐order moment $m_{i,j}$ between fMRI signals from regions i and j reflects coherent fluctuations (i.e., both activations and deactivations) in regions i and j. A positive third‐order moment $m_{i,j,k}$ between fMRI signals in regions i,j,k can reflect coherent activations in all three regions i,j,k or coherent deactivations in two of the regions (regions i and j) and simultaneous activation of the third region (region k). A positive fourth‐order moment $m_{i,j,k,l}$ between fMRI signals in regions i,j,k,l can reflect coherent fluctuations (i.e., both activations and deactivations) in all four regions or coherent fluctuations in two pairs of regions (pairs $\left(i,j\right)$ and $\left(k,l\right)$) that are anti‐correlated.

Let $x=\left(x_{1},\ldots ,x_{n}\right)$ be the fMRI activity of n brain regions at a given time‐point. We treat x as a realization of a random vector $X=\left(X_{1},\ldots ,X_{n}\right)$. Different realizations of X correspond to different time‐points. Without loss of generality, we assume that the expectation $E\left[X_{i}\right]=0$ for all brain regions i. This assumption reflects the fact that the information in fMRI signals is contained in their fluctuations, and not in their off‐sets. We refer to positive and negative deflections of $X_{i}$ as *activations* and *deactivations*, respectively. Figure 1a provides an illustration. This distinction will be important when considering higher‐order connectivity between three brain regions.

The probability distribution of X is completely determined by the moment generating function of X, which is defined as:

(1) $$M\left(\xi \right)=E\left[\exp\left(\sum_{i=1}^{n}\xi_{i}X_{i}\right)\right],$$

for $\xi =\left(\xi_{1},\ldots ,\xi_{n}\right)$. To see how the moment generating function encodes the probability distribution of X, we expand it in a Taylor series about ξ=0. This gives

(2) $$M\left(\xi \right)=\begin{array}{l} 1+\sum_{i=1}^{n}m_{i}\xi_{i}+\sum_{i,j=1}^{n}\frac{1}{2!}m_{i,j}\xi_{i}\xi_{j}+\sum_{i,j,k=1}^{n}\frac{1}{3!}m_{i,j,k}\xi_{i}\xi_{j}\xi_{k}\\ +\;\sum_{i,j,k,l=1}^{n}\frac{1}{4!}m_{i,j,k,l}\xi_{i}\xi_{j}\xi_{k}\xi_{l}+\ldots \end{array}$$

The Taylor series represents the moment generating function as a sum of monomials (the terms in Equation (2)) in the variables $\xi_{1},\ldots ,\xi_{n}$ of increasing degree. The monomial coefficients $m_{i}$, $m_{i,j}$, $m_{i,j,k}$, and so forth. are the multivariate moments of X, which will be discussed below. The subscripts i,j,k,l and so forth. take values in the set $\left\{1,2,\ldots ,n\right\}$ and correspond to brain regions. Note that there are n moments of degree one, $n^{2}$ moments of degree two and so on. Our interest, however, is in the moments that correspond to *distinct* brain regions. So, for example, we are not interested in $m_{1,1}$ and $m_{2,2}$ (i.e., the second‐order moments of regions 1 and 2) but in $m_{1,2}$ (the second‐order moment between region 1 and 2). There is a one‐to‐one correspondence between such moments of order d and subsets of d brain regions. In particular, there are $n\begin{array}{c} d \end{array}$ such moments of order d.

The coefficients of the monomials of degree one are the first‐order moments of X:

(3) $$m_{i}=\frac{\partial M}{\partial \xi_{i}}\left(0\right)=E\left[X_{i}\right]=0,$$

for i=1,…,n. In Equation (3) the term $\left(\partial M/\partial \xi_{i}\right)\left(0\right)$ denotes the partial derivative of M with respect to $\xi_{i}$ evaluated at $\xi =\left(0,\ldots ,0\right)$. The coefficients of the monomials of degree two are the second‐order moments of X:

(4) $$m_{i,j}=\frac{\partial^{2}M}{\partial \xi_{i}\partial \xi_{j}}\left(0\right)=E\left[X_{i}X_{j}\right],$$

for i,j=1,…,n. The second‐order moment $m_{i,j}$ is the covariance between the fMRI signals from regions i and j. It quantifies the extent to which the fluctuations in regions i and j are coherent, that is, co‐fluctuate. Figure 1b provides an illustration. Note that we do not have to distinguish between activations and deactivations when dealing with second‐order moments, because second‐order moments are invariant under simultaneous reversal of the signs of the signals: $E\left[\left(-X_{i}\right)\left(-X_{j}\right)\right]=E\left[X_{i}X_{j}\right]$. This, however, is not the case for third‐order moments, as explained below.

The coefficients of the monomials of degree three are the third‐order moments of X:

(5) $$m_{i,j,k}=\frac{\partial^{3}M}{\partial \xi_{i}\partial \xi_{j}\partial \xi_{k}}\left(0\right)=E\left[X_{i}X_{j}X_{k}\right],$$

for i,j,k=1,…,n. The moment $m_{i,j,k}$ quantifies the extent to which the signals from regions i,j,k co‐fluctuate. Because $m_{i,j,k}$ changes sign when the signs of all three signals are flipped, a positive third‐order moment reflects the co‐occurrence of activations, whereas a negative third‐order moment reflects the co‐occurrence of deactivations. The third‐order moment, in other words, distinguishes between activations and deactivations and this is true for all moments of odd order. However, since it is invariant under simultaneous sign reversal of two of the three signals, a positive value can also reflect the co‐deactivation of regions i and j and the simultaneous activation of region k. These two possibilities are illustrated in Figure 1b. Which interpretation is correct in a given situation can be determined by inspecting the signs of the covariances between the pairs of regions.

Finally, the coefficients of the monomials of degree four are the fourth‐order moments of X:

(6) $$m_{i,j,k,l}=\frac{\partial^{4}M}{\partial \xi_{i}\partial \xi_{j}\partial \xi_{k}\partial \xi_{l}}\left(0\right)=E\left[X_{i}X_{j}X_{k}X_{l}\right],$$

for i,j,k,l=1,…,n. The moment $m_{i,j,k,l}$ quantifies the extent to which the signals from regions i,j,k,l co‐fluctuate. Because there are an even number of regions, the fourth‐order moment is invariant under simultaneous sign flips of the signals. So, unlike third‐order moments, fourth‐order moments do not distinguish between activations and deactivations. They are also invariant under simultaneous sign flips of two of the four signals, so that positive values have two possible interpretations; either all regions co‐fluctuate with the same signs or with opposite signs. These two possibilities are illustrated in Figure 1b. Which interpretation is correct in a given situation can be assessed by inspecting the signs of the covariances between the pairs of regions.

#### 2.1.2 Multivariate cumulants of fMRI signals

Higher‐order moments cannot directly be used to infer the existence of higher‐order connectivity, because a non‐vanishing higher‐order moment might be explained by the covariances between the participating regions (Novelli & Razi, 2022). This can be understood be considering connectivity between four distinct brain regions. If each pair of regions has a positive covariance, the fourth‐order moment will be positive as well, even though there is no genuine fourth‐order connectivity between the regions. Thus, to obtain connectivity measures that are only sensitive to genuine higher‐order connectivity, the “redundant” part of the higher‐order moments needs to be removed. Here “redundant” refers to the part that can be expressed in terms of lower‐order moments. This naturally leads to the notion of multivariate cumulants. Below we will see that the non‐redundant part of a multivariate moment is the corresponding multivariate cumulant. In this section, we introduce multivariate cumulants and explain why they are natural measures of genuine higher‐order connectivity.

The cumulants of a random vector X are defined as the coefficients in the Taylor expansion of the cumulant generating function of X. The cumulant generating function is defined as the natural logarithm of the moment generating function of X:

(7) $$C\left(\xi \right)=\ln M\left(\xi \right).$$

By expanding the cumulant generating function in a Taylor series about ξ=0 we obtain a series of the following form:

(8) $$C\left(\xi \right)=\sum_{i=1}^{n}c_{i}\xi_{i}+\sum_{i,j=1}^{n}\frac{1}{2!}c_{i,j}\xi_{i}\xi_{j}+\sum_{i,j,k=1}^{n}\frac{1}{3!}c_{i,j,k}\xi_{i}\xi_{j}\xi_{k}+\sum_{i,j,k,l=1}^{n}\frac{1}{4!}c_{i,j,k,l}\xi_{i}\xi_{j}\xi_{k}\xi_{l}+\ldots$$

where the coefficients are given by the various partial derivatives of the cumulant generating function. Like the moment generating function, the cumulant generating function completely characterizes the probability distribution of X. Observe that it has the same form as the moment generating function (Equation 2): it is a linear combination of monomials in the variables $\xi_{1},\ldots ,\xi_{n}$. The coefficients $c_{i}$ are referred to as the *first‐order cumulants* of X, $c_{i,j}$ are the *second‐order cumulants* of X, and so on. Note that there is a one‐to‐one correspondence between the moments and cumulants of X of any given order.

To see why cumulants are natural measures of genuine higher‐order connectivity, we express them in terms of moments. The first‐order cumulant of region i is equal to the first‐order moment of region i:

(9) $$c_{i}=\frac{\partial C}{\partial \xi_{i}}\left(0\right)=m_{i}.$$

The second‐order cumulant between regions i and j is:

(10) $$c_{i,j}=\frac{\partial^{2}C}{\partial \xi_{i}\partial \xi_{j}}\left(0\right)=m_{i,j}-m_{i}m_{j}.$$

So $c_{i,j}$ is the covariance between $X_{i}$ and $X_{j}$. Equation (10) shows that a second‐order cumulant vanishes if and only if the corresponding second‐order moment can be expressed in terms of moments of lower order (in this case of order one): $m_{i,j}=m_{i}m_{j}$. Functionally, $c_{i,j}=0$ means that $m_{i,j}$ is “explained” by the first‐order moments $m_{i}$ and $m_{j}$, and is in this sense redundant. Thus, the second‐order cumulant $c_{i,j}$ is the non‐redundant part of the second‐order moment $m_{i,j}$. Now consider the third‐order cumulant $c_{i,j,k}$. It can be expressed in terms of moments as:

(11) $$c_{i,j,k}=\frac{\partial^{3}C}{\partial \xi_{i}\partial \xi_{j}\xi_{k}}\left(0\right)=m_{i,j,k}-m_{i}m_{j,k}-m_{j}m_{i,k}-m_{k}m_{i,j}.$$

Equation (11) shows that the third‐order cumulant vanishes if and only if the corresponding third‐order moment $m_{i,j,k}$ can be expressed in terms of moments of lower order:

(12) $$m_{i,j,k}=m_{i}m_{j,k}+m_{j}m_{i,k}+m_{k}m_{i,j}.$$

Functionally, $c_{i,j,k}=0$ means that the third‐order moment $m_{i,j,k}$ can be “explained” by the first‐ and second‐order moments and is in this sense redundant.

Since we have assumed that $m_{i}=0$, Equations ((9), (10), (11)) simplify to $c_{i}=0$, $c_{i,j}=m_{i,j}$, and $c_{i,j,k}=m_{i,j,k}$, respectively, and hence show that the cumulants up to and including order three are identical to the corresponding moments. This is not true for the fourth‐order cumulant, which can be expressed in terms of moments as:

(13) $$c_{i,j,k,l}=\frac{\partial^{4}C}{\partial \xi_{i}\partial \xi_{j}\xi_{k}\xi_{l}}\left(0\right)=m_{i,j,k,l}-m_{i,j}m_{k,l}-m_{i,k}m_{j,l}-m_{i,l}m_{j,k}.$$

Equation (13) shows that the fourth‐order cumulant vanishes if and only if the corresponding fourth‐order moment $m_{i,j,k,l}$ can be expressed in terms of lower‐order moments:

(14) $$m_{i,j,k,l}=m_{i,j}m_{k,l}+m_{i,k}m_{j,l}+m_{i,l}m_{j,k}.$$

So, the fourth‐order cumulant measures the non‐redundant part of the fourth‐order moment.

#### 2.1.3 Higher‐order connectivity measures

In Section 2.1.2, we explained that multivariate cumulants, but not multivariate moments, are measures of genuine higher‐order connectivity. In the current section we use third‐ and fourth‐order cumulants to construct third‐ and fourth‐order connectivity measures. In the statistics literature, these measures are referred to as the *coskewness* and *cokurtosis*, respectively, and they are obtained from the third‐ and fourth‐order cumulants by appropriate normalization. We will also adjust the edge connectivity measure proposed in (Faskowitz et al., 2020) to render it a measure of genuine fourth‐order connectivity.

To obtain dimensionless measures, the moments that appear in the definition of the cumulants are divided by the product of the standard deviations of the fMRI signals of the participating brain regions. The normalized moments are commonly referred to as *correlations* and we will denote them by $r_{i,j}$, $r_{i,j,k}$, $r_{i,j,k,l}$, and so forth. They are hence given by:

(15) $$r_{i,j}=\frac{m_{i,j}}{\sigma_{i}\sigma_{j}},r_{i,j,k}=\frac{m_{i,j,k}}{\sigma_{i}\sigma_{j}\sigma_{k}},r_{i,j,k,l}=\frac{m_{i,j,k,l}}{\sigma_{i}\sigma_{j}\sigma_{k}\sigma_{l}},$$

where $\sigma_{i}^{2}=m_{\mathrm{ii}}$ is the variance of the fMRI signal in region i. The corresponding normalized cumulants are denoted by $r_{i,j}^{c}$, $r_{i,j,k}^{c}$, $r_{i,j,k,l}^{c}$, and so forth:

(16) $$r_{i,j}^{c}=\frac{c_{i,j}}{\sigma_{i}\sigma_{j}},r_{i,j,k}^{c}=\frac{c_{i,j,k}}{\sigma_{i}\sigma_{j}\sigma_{k}},r_{i,j,k,l}^{c}=\frac{c_{i,j,k,l}}{\sigma_{i}\sigma_{j}\sigma_{k}\sigma_{l}},$$

So, the redundant and non‐redundant correlations are the normalized moments and cumulants, respectively. In quantum field theory, the redundant and non‐redundant correlations are referred to as *disconnected* and *connected*, respectively (hence the superscript “c”) (Schweigler et al., 2017).

Note that the second‐order non‐redundant correlation is the Pearson correlation coefficient. Correlations between more than two regions are referred to as *higher‐order*. They are used in many scientific fields, including quantum mechanics (Schweigler et al., 2017), cosmology (Takada & Jain, 2003), and statistical thermodynamics (Jensen, 2012), to study higher‐order interactions in physical systems. Only the second‐order correlation is confined to the interval $\left[-1,1\right]$. However, due to the normalization, the correlations of any order are dimensionless and can therefore be compared across data‐sets, subjects, and studies. In the statistical literature, the third‐ and fourth‐order non‐redundant correlations are referred to as (excess) *coskewness* and (excess) *cokurtosis*, respectively. They are multivariate generalizations of the notions of skewness and kurtosis. In this study, due to computational constraints, we will only consider higher‐order correlations up to and including order four, that is, the coskewness and the cokurtosis.

Besides coskewness and cokurtosis, we also consider a recently proposed fourth‐order connectivity measure referred to as the *edge connectivity* (Faskowitz et al., 2020). The edge connectivity is a special case of the measure proposed earlier in Martellini and Ziemann (2010). Like higher‐order moments, the edge connectivity cannot be used directly for assessing higher‐order connectivity, because it has a redundant part (Novelli & Razi, 2022). We can, however, adjust it to obtain a non‐redundant measure as described below. The *edge connectivity* between edges $\left(i,j\right)$ and $\left(k,l\right)$ is defined as:

(17) $$\varepsilon_{\mathrm{ij},\mathrm{kl}}=\frac{m_{i,j,k,l}}{{\left(m_{i,i,j,j}m_{k,k,l,l}\right)}^{1/2}},$$

and takes values in the interval $\left[-1,1\right]$ (Faskowitz et al., 2020). To see that it contains a redundant part, we use Equation (13) to express the edge connectivity in terms of cumulants:

(18) $$\varepsilon_{\mathrm{ij},\mathrm{kl}}=\frac{c_{i,j,k,l}+m_{i,j}m_{k,l}+m_{i,k}m_{j,l}+m_{i,l}m_{j,k}}{{\left(\left(c_{i,i,j,j}+2m_{i,j}^{2}+m_{i,i}m_{j,j}\right)\left(c_{k,k,l,l}+2m_{k,l}^{2}+m_{k,k}m_{l,l}\right)\right)}^{1/2}}.$$

The redundant part $\varepsilon_{\mathrm{ij},\mathrm{kl}}^{r}$ is obtained by setting the fourth‐order cumulants to zero. This gives,

(19) $$\varepsilon_{\mathrm{ij},\mathrm{kl}}^{r}=\frac{m_{i,j}m_{k,l}+m_{i,k}m_{j,l}+m_{i,l}m_{j,k}}{{\left(\left(2m_{i,j}^{2}+m_{i,i}m_{j,j}\right)\left(2m_{k,l}^{2}+m_{k,k}m_{l,l}\right)\right)}^{1/2}},$$

The non‐redundant part of the edge connectivity is now obtained by subtracting the redundant part:

(20) $$\varepsilon_{\mathrm{ij},\mathrm{kl}}^{c}=\varepsilon_{\mathrm{ij},\mathrm{kl}}-\varepsilon_{\mathrm{ij},\mathrm{kl}}^{r}.$$

In the remainder of the text, we will refer to the corrected edge connectivity $\varepsilon_{\mathrm{ij},\mathrm{kl}}^{c}$ simply as “edge connectivity.”

### 2.2 Estimation and inference

#### 2.2.1 Estimation

Let $X^{\left(1\right)},\ldots ,X^{\left(T\right)}$ be n‐dimensional random vectors that model the fMRI activity in n brain regions at T points in time. The i‐th coordinate of $X^{\left(t\right)}$ is denoted by $X_{i}^{\left(t\right)}$ for t=1,…,T. Thus, $X_{i}^{\left(t\right)}$ is the value of the fMRI signal in region i and time t. We assume that the fMRI signals have been z‐scored so that they have sample mean zero:

$$\frac{1}{T}\sum_{t=1}^{T}X_{i}^{\left(t\right)}=0,$$

for i=1,…,n. We furthermore assume that the signals have been normalized to unit variance:

$$\frac{1}{T-1}\sum_{t=1}^{T}{\left(X_{i}^{\left(t\right)}\right)}^{2}=1$$

for i=1,…,n. No information is lost in this normalization, because the connectivity measures are dimensionless. The normalization only serves to simplify the measures' formulas.

Since the connectivity measures described in Section 2.1.3 can be expressed in terms of moments of the fMRI signals, the most straightforward way to estimate the measures is to replace the moments by sample moments. In other words, the expectation operator that appears in the definition of the moments is replaced by (temporal) averaging. Thus, the estimators of the second and third‐order correlations are:

(21) $${\hat{r}}_{i,j}=\frac{1}{T}\sum_{t=1}^{T}X_{i}^{\left(t\right)}X_{j}^{\left(t\right)},$$

and

(22) $${\hat{r}}_{i,j,k}=\frac{1}{T}\sum_{t=1}^{T}X_{i}^{\left(t\right)}X_{j}^{\left(t\right)}X_{k}^{\left(t\right)},$$

respectively, and so on for higher orders. Note that the index p runs over the N observations $X^{\left(1\right)},\ldots ,X^{\left(N\right)}$. Such estimators are referred to as *plug‐in estimators*. The plug‐in estimators for the correlations are used to construct estimators ${\hat{r}}_{i,j}^{c}$, ${\hat{r}}_{i,j,k}^{c}$, and ${\hat{r}}_{i,j,k,l}^{c}$ for the correlation $r_{i,j}^{c}$, coskewness $r_{i,j,k}^{c}$, and cokurtosis $r_{i,j,k,l}^{c}$, respectively, as well as an estimator ${\hat{\varepsilon }}_{\mathrm{ij},\mathrm{kl}}^{c}$ for the (non‐redundant) edge connectivity $\varepsilon_{\mathrm{ij},\mathrm{kl}}^{c}$.

In Appendices A and B, we derive the asymptotic sampling distributions of the plug‐in estimators of the coskewness, cokurtosis, and edge connectivity, under the assumption that the observations at different time‐points are independent. The asymptotic distributions make clear that the estimators are asymptotically unbiased and normally distributed and can in principle be used to construct (asymptotic) confidence intervals and hypothesis tests for the higher‐order connectivity measures. Unfortunately, although N might be sufficiently large for the confidence intervals and hypothesis tests to be valid, they require the observations to be independent, which typically is not the case in practice since fMRI signals have (positive) auto‐correlations. Consequently, the confidence intervals derived from the asymptotic distributions will be too narrow and the null distributions will have too small variances, giving rise to spurious connectivity. In practice, therefore, we need to use randomization techniques to carry out statistical inference.

#### 2.2.2 Statistical inference

The presence of auto‐correlations in fMRI data complicates statistical inference about higher‐order correlations from individual subjects, because for such data, the null and sampling distributions of the plug‐in estimators are unknown. This makes drawing conclusions from such data considerably more challenging than in the case of group data, because for group data, the sampling distributions can be approximated by using independent bootstrapping over subjects, at least if sufficiently many subjects are available (Efron & Tibshirani, 1986). If, in addition to multiple subjects, contrasting conditions are available, either z‐ or t‐tests can be used or the null distributions can be approximated using permutation tests or independent bootstrapping. In the present study, we focus on inference from single‐subject data, which requires the use of randomization techniques to take into account auto‐correlations in the fMRI signals. In general, hypothesis testing can be done either by approximating the null or the sampling distribution of a given statistic, the latter of which also allows for the construction of confidence intervals. We first discuss randomization techniques for approximating null distributions for hypothesis testing about higher‐order connectivity that have recently been proposed.

As mentioned in Section 1, different fMRI studies use different randomization techniques and there is some discussion on which technique is most appropriate (Betzel et al., 2022; Jo et al., 2021). This issue might be clarified by making explicit the null hypotheses that correspond to the different randomization techniques. The most widely used technique to test the null hypothesis of no (time‐resolved) higher‐order interaction, involves testing for large‐amplitude fluctuations in the multiple edge time‐series (Betzel et al., 2022). Null‐data are constructed by circularly shifting each time‐series by a random number of samples. This preserves the signal means, their variances, and approximately their auto‐correlation functions. It also retains any non‐stationarities that might be present in the signals. However, it largely removes any cross‐correlations between different time‐series and does this entirely if the shifts are larger than the characteristic time‐scale of the signals' auto‐correlations. The null‐hypothesis corresponding to this randomization technique, therefore, is that the signals are uncorrelated. Hence, rejection of the null hypothesis means that the observed value of the edge connectivity cannot be explained by uncorrelated signals. In other words, if the null hypothesis is rejected, we can conclude that the signals are uncorrelated. This obviously is not the intended conclusion and hence this kind of randomization is not appropriate for hypothesis testing for higher‐order connectivity.

Randomization techniques that are more appropriate for testing for higher‐order connectivity are coherent phase‐randomization (Prichard & Theiler, 1994) and auto‐regressive randomization (Saggar et al., 2022) because they take into account the auto‐ and cross‐correlation structure within the data. Both techniques generate Gaussian null‐data and hence correspond to the null‐hypothesis that all higher‐order connectivities are zero. Although this null‐hypothesis is stronger than the intended one, which is that only the third‐order (or fourth‐order) connectivity is zero, their use in combination with a third‐order (or fourth‐order) connectivity measure as test‐statistic, gives at least some confidence for the presence of third‐order (or fourth‐order) connectivity. A drawback of these techniques is that they rely on the assumption of linearity and stationarity, so that a rejection of the null‐hypothesis might reflect the presence of higher‐order connectivity, but also that of non‐linearity, non‐stationarity, or a combination of these. Another drawback is that these techniques, by their very nature, cannot be used for the construction of confidence intervals. For these reasons, in this study we explore the use of bootstrap techniques, which can in principle be used both for testing and for constructing confidence intervals.

Let X be a n×T data matrix comprising n simultaneously recorded fMRI time‐series of length T and let $T\left(X\right)$ be an estimator of a parameter θ. If the sampling distribution of $T\left(X\right)$ is known, it can be used to construct confidence intervals for θ and to test hypothesis about θ. If the sampling distribution of $T\left(X\right)$ does not depend on the auto‐ and cross‐correlation structure within the data, its sampling distribution can be approximated by bootstrapping individual observations. That is, one randomly draws T columns from X with replacement, combines them into a n×T bootstrap data matrix $X^{*}$ and computes $T\left(X^{*}\right)$. By repeating this a large number of times, an approximation of the sampling distribution of $T\left(X\right)$ is obtained. This is referred to as the independent bootstrap (Efron & Tibshirani, 1986). The sampling distributions of the estimators of most parameters of interest, however, depend on the auto‐ and cross‐correlation structure within the fMRI signals, so that the independent bootstrap is of limited use in this context.

The most straightforward bootstrap technique that takes into account the auto‐ and cross‐correlation structure within the data is the block bootstrap (Kunsch, 1989) which involves sampling blocks of consecutive observations, rather than individual observations. Thus, the fMRI signals are divided into non‐overlapping blocks of a fixed length L that divides T and the fMRI signals are cut into B=T/L non‐overlapping blocks of length L. We then sample B blocks with replacement and concatenate them to obtain a bootstrapped data matrix $X^{*}$ from which we compute the statistic $T\left(X^{*}\right)$. By repeating this a large number of times, we obtain an approximation of the sampling distribution of $T\left(X\right)$.

A related technique is the stationary bootstrap (Politis & Romano, 1994) which yields stationary time‐series. If the observations are stationary and have short‐range auto‐ and cross‐correlations, both techniques are asymptotically valid for a large class of statistics. This means that the approximated standard errors converge to the true ones if sufficient data is available. However, it is unclear how they perform on finite data sets with dependencies encountered in resting‐state fMRI signals. This is an empirical question that is best assessed by using simulated data, which we will do in this study. We focus on the block bootstrap because the stationary bootstrap yielded practically identical results.

### 2.3 Generative model

#### 2.3.1 Model description

To assess the performance of the connectivity measures and the block bootstrap, we generated synthetic fMRI signals using a generative model. We used a simple model so that explicit formulas for the connectivity measures can be derived. These formulas serve as the ground‐truth and are used to assess the quality of the connectivity measures.

Let $X^{\left(t\right)}\in {\mathbb{R}}^{n}$ be the t‐th sample of the fMRI activity vector of n brain regions. The activity of the i‐th brain region is denoted by $X_{i}^{\left(t\right)}$. We will only be concerned with the cases n=3 (for modeling third‐order connectivity) and n=4 (for modeling fourth‐order connectivity). The model needs to be sufficiently flexible to allow for arbitrary correlations between the fMRI signals that can be chosen independently of the time‐scale of the auto‐correlations and the values of the higher‐order moments.

We model $X_{i}^{\left(t\right)}$ as a first‐order auto‐regressive process that is driven by the sum of a Gaussian and a non‐Gaussian process:

(23) $$X_{i}^{\left(t+1\right)}=\phi X_{i}^{\left(t\right)}+Z_{i}^{\left(t\right)}+\psi U^{\left(t\right)}.$$

In Equation (23) $Z_{i}$ is a zero‐mean and unit‐variance white Gaussian processes. The parameter 0≤ϕ<1 controls the time‐scale of the auto‐correlations and ψ≥0 controls the contribution of the non‐Gaussian process U to the simulated fMRI signal. Note that for each region the same random variable U is added. This will give rise to third‐order correlations in X as described below.

$U^{\left(t\right)}$ can be any (zero‐mean and unit‐variance) non‐Gaussian random variable and an appropriate choice depends on which higher‐order connectivities are of interest. For example, and as explained in more detail later on, in assessing the performance of the coskewness, which relates to multivariate asymmetry, we let U follow an asymmetric distribution (the skew‐normal distribution). Likewise, in assessing the performance of the cokurtosis and (non‐redundant) edge connectivity, we let U follow a heavy‐tailed distribution (the t‐distribution). For simplicity we assume that the correlation between $Z_{i}$ and $Z_{j}$ equals a constant –1 < ρ < 1 for all i≠j. The characteristic time‐scale of the auto‐correlations in $X_{i}$ is $-\ln\left(\phi \right)$ samples, so to ensure a characteristic time‐scale of τ samples we set $\phi =\exp\left(-\tau \right)$.

We now obtain a ground‐truth expression for the correlation $r_{\mathrm{ij}}^{c}$. The covariance between each pair $\left(i,j\right)$ of brain regions is $\left(\rho +\psi^{2}\right)/\left(1-\phi^{2}\right)$ and the variance of each region is $\left(1+\psi^{2}\right)/\left(1-\phi^{2}\right)$ and therefore, the correlation between each pair $\left(i,j\right)$ of brain regions is:

(24) $$r_{i,j}^{c}=\frac{\rho +\psi^{2}}{1+\psi^{2}}.$$

To ensure that the correlation between any two fMRI signals is equal to some desired value r, we therefore need to set

(25) $$\rho =r+\left(r-1\right)\psi^{2}.$$

However, because the covariance matrix of $E\left(t\right)$ needs to be positive‐definite, not all values of r are possible, but only those for which $\rho >-1/\left(n-1\right)$. This follows from the fact that the eigenvalue $1+\left(n-1\right)\rho$ of the covariance matrix of E needs to be positive. This requirement implies that

(26) $$r>\frac{\psi^{2}}{\psi^{2}+1}-\frac{1}{\left(\psi^{2}+1\right)\left(n-1\right)}.$$

We will restrict ψ to the interval $\left[0,1\right]$ and because the function on the right‐hand‐side of Equation (24) is increasing in ψ, it is sufficient that

(27) $$r>\frac{1}{2}-\frac{1}{2\left(n-1\right)}.$$

In particular, for n=3 and n=4 we need r>1/4 and r>1/3, respectively, which are realistic values for resting‐state fMRI signals.

#### 2.3.2 General expressions for higher‐order connectivities

We now derive expressions for the coskewness, cokurtosis, and edge connectivity in terms of the parameters of the generative model. Besides the parameters ϕ and ψ, the expressions depend on the probability distribution of the random variable U. We do not make assumptions about U except that it has finite third‐ and fourth‐order cumulants. The expressions for the coskewness, cokurtosis, and edge connectivity will involve these cumulants.

We denote the third‐ and fourth‐order cumulants of U by $c_{3}^{U}$ and $c_{4}^{U}$, respectively. The coskewness between any triplet of brain regions i,j,k is then given by,

(28) $$r_{i,j,k}^{c}=\frac{{\left(1-\phi^{2}\right)}^{3/2}\psi^{3}}{\left(1-\phi^{3}\right){\left(1+\psi^{2}\right)}^{3/2}}c_{3}^{U}$$

and the cokurtosis between any quadruple of brain regions i,j,k,l is:

(29) $$r_{i,j,k,l}^{c}=\frac{{\left(1-\phi^{2}\right)}^{2}\psi^{4}}{\left(1-\phi^{4}\right){\left(1+\psi^{2}\right)}^{2}}c_{4}^{U}.$$

Note that the coskewness and cokurtosis do not depend on the correlation ρ and hence also not on the correlation between the signals from different brain regions.

An expression for the non‐redundant connectivity between edges $\left(i,j\right)$ and $\left(k,l\right)$ can be obtained from its definition (Equations 20 and 29) which gives

(30) $$\varepsilon_{\mathrm{ij},\mathrm{kl}}=\frac{\psi^{4}\left(c_{4}^{U}+3\right)+3\rho^{2}+6\phi^{2}\left(\rho +\psi^{2}\right)/\left(1-\phi^{2}\right)+6{\rho \psi }^{2}}{1+2\rho^{2}+\psi^{4}\left(c_{4}^{U}+3\right)+2\left(1+\rho \right)\psi^{2}+2\phi^{2}\left({\left(1+\psi^{2}\right)}^{2}+2{\left(\rho +\psi^{2}\right)}^{2}\right)/\left(1-\phi^{2}\right)}.$$

Its redundant part is obtained by setting $c_{4}^{U}=0$ in Equation (30). Note that, in contrast to the coskewness and cokurtosis, the (non‐redundant) edge connectivity depends on ρ and hence on the (common) correlation between the signals from different brain regions.

#### 2.3.3 Modeling third‐order connectivity

To generate synthetic fMRI signals that exhibit third‐order connectivity, we need to make a concrete choice for the probability distribution of the random variable U that appears in the generative model. This will yield concrete formulas for the coskewness as explained below.

For simulating third‐order connectivity we let n=3 in the generative model and assume that the non‐Gaussian fluctuations U follow a skew‐normal distribution. The skew‐normal distribution is specified by a mean μ, a variance $\kappa^{2}$, and a shape parameter α that controls the skewness. In particular, α=0 corresponds to the normal distribution with mean μ and variance $\sigma^{2}$ and positive/negative values of α correspond to a right−/left‐skew, respectively. Let:

(31) $$\delta =\frac{\alpha }{\sqrt{1+\alpha^{2}}}.$$

The mean and variance of U can be expressed in terms of δ as:

(32) $$c_{U}^{1}=\mu +\kappa \delta \sqrt{\frac{2}{\pi }},$$

and

(33) $$c_{U}^{2}=\left(1-\frac{2\delta^{2}}{\pi }\right)\kappa^{2},$$

respectively. So to ensure that U has zero mean and unit variance we need to take the following values for the parameters μ and σ:

(34) $$\kappa^{2}=\frac{\pi }{\pi -2\delta^{2}}$$

and

(35) $$\mu =-\kappa \delta \sqrt{\frac{2}{\pi }}.$$

The third‐order cumulant of U is:

(36) $$c_{U}^{3}=\frac{4-\pi }{2}\frac{{\left(\delta \sqrt{2/\pi }\right)}^{3}}{{\left(1-2\delta^{2}/\pi \right)}^{3/2}}.$$

Substituting Equations (36) into (28) gives the formula for the coskewness between any three brain regions i,j,k:

(37) $$r_{i,j,k}^{c}=\frac{{\left(1-\phi^{2}\right)}^{3/2}\psi^{3}}{\left(1-\phi^{3}\right){\left(1+\psi^{2}\right)}^{3/2}}\frac{4-\pi }{2}\frac{{\left(\delta \sqrt{2/\pi }\right)}^{3}}{{\left(1-2\delta^{2}/\pi \right)}^{3/2}}.$$

A plot of $c_{U}^{3}$ as a function of δ shows that $c_{U}^{3}$ increases from 0 to nearly 1 so that the maximal value of the coskewness is approximately $\psi^{3}/{\left(1+\psi^{2}\right)}^{3/2}$.

To obtain realizations of the random variable U we use the fact that it has the following stochastic representation:

(38) $$U=-\sigma_{U}\delta \sqrt{\frac{2}{\pi }}+\sigma_{U}\left(\delta |Z_{1}|+\sqrt{1-\delta^{2}}Z_{2}\right),$$

where $Z_{1}$ and $Z_{2}$ are independent and standard‐normally distributed and $\sigma_{U}=\sqrt{\pi /\left(\pi -2\delta^{2}\right)}$ (Henze, 1986).

#### 2.3.4 Modeling fourth‐order connectivity

To generative synthetic fMRI signals that exhibit fourth‐order connectivity, we need to make a concrete choice for the probability distribution of the random variable U that appears in the generative model. This will yield concrete formulas for the cokurtosis and edge connectivity as explained below.

For simulating fourth‐order connectivity we let n=4 in the generative model and assume that the non‐Gaussian fluctuations U follow a t‐distribution with unit variance:

(39) $$U=\sqrt{\frac{\nu -2}{\nu }}U^{\prime },$$

where $U^{\prime }$ follows a t‐distribution with ν degrees of freedom. The normalization constant ensures that U has unit variance. Because $c_{U}^{4}=6/\left(\nu -4\right)$, the cokurtosis between any four brain regions i,j,k,l is only defined for ν≥5 and is given by:

(40) $$r_{i,j,k,l}^{c}=\frac{{\left(1-\phi^{2}\right)}^{2}\psi^{4}}{\left(1-\phi^{4}\right){\left(1+\psi^{2}\right)}^{2}}\frac{6}{\nu -4},$$

which is obtained by substituting the expression for $c_{U}^{4}$ into Equation (29).

Since $m_{U}^{4}=3\left(\nu -2\right)/\left(\nu -4\right)$, the non‐redundant edge connectivity between any two edges $\left(i,j\right)$ and $\left(k,l\right)$ is obtained by substituting the expression for $m_{4}^{U}$ into Equation (30). Note that the edge connectivity is only defined for ν≥5.

### 2.4 BOLD‐fMRI data‐sets and pre‐processing

#### 2.4.1 Human connectome project

We used the resting‐state BOLD‐fMRI data from the 94 subjects of the Human Connectome Project that also underwent magnetoencephalographic recordings (Glasser et al., 2013; Glasser, Smith, et al., 2016; Larson‐Prior et al., 2013). We used these subjects in order to compare the results with those obtained from MEG data in a subsequent study. Each subject was scanned four times using gradient‐echo echo‐planar imaging with a 3T Siemens Connectome Skyra scanner for 15 min. The subjects were asked to lie still and fixate at a white cross‐hair on a dark background, think of nothing in particular, and not to fall asleep. This yielded 1200 volumes with a cubic resolution of 2 mm and a repetition time of 0.72 s. The data were subsequently registered to standard HCP cortical meshes. Nuisance signals such as motion‐derived artifacts and physiological noise were cleaned using ICA‐FIX, a classifier approach that removes “bad” components from the data. These procedures yield the FIX‐denoized‐compact HCP data (Smith et al., 2013). Prior to analysis, the fMRI signals were bandpass filtered between 0.01 and 0.1 Hz using a zero‐phase fourth‐order Butterworth filter and subsequently averaged over the regions‐of‐interest in the Glasser parcellation (Glasser, Coalson, et al., 2016).

#### 2.4.2 Clinical sample: Multiple sclerosis

We used resting‐state BOLD‐fMRI from 330 people with MS and 95 healthy controls from the Amsterdam MS cohort (as described previously in Broeders et al., 2022, Eijlers et al., 2017, Meijer et al., 2017, Schoonheim et al., 2021, Strik et al., 2021). Clinically definite MS was established in accordance with the 2010 revised McDonald criteria (Polman et al., 2011). The patients were relapse‐free and were not under steroid treatment for 2 months or more before participating in the study. Additionally, they had no history of another psychiatric or neurological disease. All participants provided written informed consent forms and the appropriate approval was obtained from the institutional ethics review board.

Cognitive assessment was performed using the brief repeatable battery of neuropsychological tests (Rao, 1990). Test scores were combined to form seven cognitive domains: executive functioning, verbal memory, information processing speed, verbal fluency, visuospatial memory, working memory, and attention. Patients were classified as cognitively impaired (CI) if they scored 2 standard deviations or more below controls on at least two cognitive domains, as mildly CI (MCI) if they scored between 1.5 and 2 standard deviations below controls on two or more cognitive domains, and the remaining patients were considered cognitively preserved (CP). Finally, average cognition was determined for all individuals by averaging scores of all domains.

MRI was performed with a 3T GE “Signa‐HDxt” MRI (Wilwaukee, WI) using an 8‐channel phased‐array head coil. A 3D T1‐weighted fast spoiled gradient echo sequence was obtained for accurate segmentation of brain regions. A 3D T2‐weighted fluid‐attenuated inversion recovery (FLAIR) scan was acquired for segmentation of white‐matter lesions in MS patients. Finally, an echo planar imaging (EPI) sequence was performed to acquire resting‐state fMRI scan, during which participants were asked to lie with their eyes closed and think of nothing in particular. This yielded 202 volumes with a resolution of 3.3×3.3×3 mm^3^ and a repetition time of 2.2 s.

Pre‐processing of the functional MRI data has been updated since the previous analysis. In short, structural image pre‐processing consisted of white‐matter lesion segmentation and filling (for patients), brain extraction, brain tissue segmentation (white‐, grey‐matter, and cerebrospinal fluid [CSF]), non‐linear registration to standard space, and finally segmentation of brain regions using cortical regions from the Brainnetome atlas (Fan et al., 2016) and deep grey matter regions using FSL's FIRST (Patenaude et al., 2011). Functional pre‐processing involved removing the first two dummy scans, motion and slice‐time correction, brain extraction, Gaussian spatial smoothing, motion artefact removal using ICA‐AROMA (Pruim et al., 2015), regression‐based removal of mean white‐matter and CSF signal, high‐pass temporal filtering (0.02 Hz), and the final steps included boundary‐based linear registration to the T1‐weighted image.

Cortical and deep grey matter regions were registered to the functional images and used to create time‐series for all brain regions. All cortical brain regions (Fan et al., 2016) were assigned to one of six cortical networks based on maximum overlap (Yeo et al., 2011): the DMN, frontoparietal network (FPN), dorsal attention network, ventral attention network, visual network, and sensorimotor network. Finally, all deep grey matter regions were combined into a distinct network.

Intra‐DMN connectivity was calculated by averaging the absolute second‐order correlations (Pearson correlation) between all regions assigned to the DMN. Extra‐DMN connectivity was computed for each of the six other networks by averaging the absolute second‐order correlation between regions of these networks. For third‐ and fourth‐order connectivity, it was possible to have more than one node in either of the two networks. For instance, third‐order connectivity between the DMN and FPN can encompass one DMN node and two FPN nodes or vice versa. We determined the multicollinearity between these combinations using a simple regression model with average cognition as dependent variable and if the variable inflation factor (VIF) reached above 10 we used the mean over these combinations in subsequent analyses.

Pairwise connectivity values are often normalized using the global connectivity value, as it is thought to remove noise and improve connectome “fingerprinting” (Finn et al., 2015). However, this has never been determined for higher‐order connectivity. Therefore, we chose to not normalize pairwise connectivity using global connectivity, but to include global connectivity as covariate in all statistical models. For pairwise and third‐order connectivity, the mean absolute connectivity strength between all brain regions was computed. This was not feasible for all fourth‐order connections, so 10,000 random quadruplets were selected and the mean connectivity strength of those quadruplets was included as global fourth‐order connectivity.

Statistical analyses were performed in IBM SPSS version 28 (Armonk, NY, USA) and *p*‐values <.05 were considered statistically significant. We checked normality of all connectivity values using the Kolmogorov–Smirnov test and by histogram inspection, with log‐transformations being applied to all higher‐order connectivity metrics as they did not pass these tests. All analyses were corrected for age, sex, level of education and global connectivity (of the corresponding order). The level of education was based on the highest level that was attained, these scores were binarized for all analyses (higher professional education yes/no). We compared within‐DMN and extra‐DMN pairwise connectivity across cognitive groups (HC, CP, MCI and CI) using linear mixed models. The p‐values were adjusted for performing multiple comparisons using Bonferroni (both unadjusted and adjusted values are reported: *p* and *p*‐adj). If a difference between groups was observed for pairwise connectivity, the same network pair was compared across groups for third and fourth‐order connectivity as well. Finally, we performed a multiple linear regression on data from MS patients with average cognition as a dependent variable, to see if higher‐order connectivity of the DMN explains additional variance beyond pairwise connectivity. For this regression analysis, we included all variables that showed significant group differences.

## 3 RESULTS

### 3.1 Simulations

#### 3.1.1 Higher‐order connectivity in simulated fMRI data

To assess the performance of the plug‐in estimators for the coskewness, cokurtosis, and edge connectivity, we use synthetic fMRI signals obtained by sampling from the generative model described in Section 2.3. To obtain appropriate values for the model parameters, we need to know how the true higher‐order connectivity in the model depends on the model parameters. This issue will be considered in this section. In the model, all pairs of fMRI signals have the same correlation r and the auto‐correlations of each signal have a characteristic time‐scale τ. We first consider appropriate values for r and τ.

To obtain a realistic value for the time‐scale τ, we estimated the auto‐correlation functions of the fMRI signals of all cortical regions and subjects and averaged them across regions and then across subjects (HCP dataset). Since the auto‐correlation function of the model has the form $\exp\left(-k/\tau \right)$, where τ denotes the lag in number of samples, we fitted this function to the group‐level auto‐correlation function of the fMRI data using least squares. This gave an optimal value of τ=2 samples. With a repetition time of 0.72 s, this corresponds to a time‐scale of 1.44 s. As discussed in Section 2.3.1, the correlation between the simulated fMRI signals needs to exceed 1/4 (in the case of third‐order connectivity) and 1/3 (in the case of fourth‐order connectivity) for the model to be well‐defined. We therefore set r=0.4, which, although a bit high, is still within the experimental range. These values for r and τ were used in all simulations.

We now analyze the behavior of the true higher‐order connectivity as a function of the model parameters. We first consider third‐order connectivity. The generative model for fMRI signals with third‐order connectivity, as measured by the coskewness, is a three‐dimensional auto‐regressive process with Gaussian as well as non‐Gaussian innovations (Equation (23)). It models the joint dynamics of the fMRI signals in three brain regions. The strength (i.e., standard‐deviation) of the non‐Gaussian innovations is controlled by a parameter 0≤ψ≤1, where ψ=0 corresponds to strength zero, i.e., no non‐Gaussian innovations, and for ψ=1 the Gaussian and non‐Gaussian innovations are equally strong. The non‐Gaussian innovations follow a skew‐normal distribution with parameter α, which controls the skewness of the distribution. Thus, α=0 corresponds to the normal distribution and for positive values of α, the distribution is right‐skewed, which means that extreme activations are more likely than extreme deactivations. Because all three regions receive the same non‐Gaussian innovations, they have the tendency to simultaneously activate, but not deactivate and, as such, engage in third‐order interactions, which is reflected in a positive coskewness (see Figure 1a). For negative values of α, (simultaneous) extreme deactivations are more likely than extreme activations. However, since these two cases are mathematically equivalent, we only consider the case α≥0.

**FIGURE 2:**
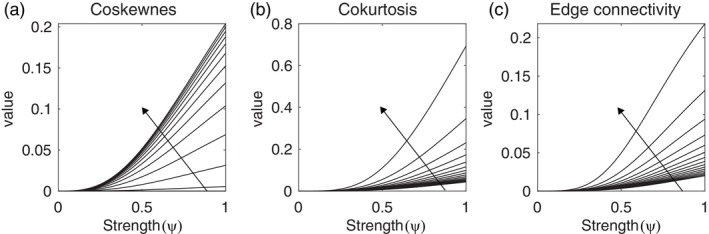
Higher‐order connectivity in simulated functional magnetic resonance imaging (fMRI) data. (a) Coskewness as a function of the strength ψ of the non‐Gaussian innovations. The curves correspond to different values of the shape parameter α, which ranged from 0 to 6 in step of 0.5. The arrow points in the direction of increasing α. (b) Cokurtosis as a function of the amplitude ψ of the non‐Gaussian innovations. The curves correspond to different values of the parameter ν, which ranged from 5 to 20 in steps in 1. The arrow points in the direction of decreasing ν. (c) Same format as (b) but for the non‐redundant edge connectivity.

Figure 2a shows the coskewness as a function of ψ for different values of α. For α=0 the simulated fMRI signals are Gaussian and hence their coskewness is zero. Their coskewness increases for increasing values of α and converges towards a maximum coskewness of about 0.2 for ψ=1. Further increasing α does not increase the coskewness. In the simulations we therefore let α range between 0 and 3.

The generative model for fMRI signals with fourth‐order connectivity, as measured by the cokurtosis or edge connectivity, is a four‐dimensional auto‐regressive process with Gaussian and non‐Gaussian innovations. It models the joint dynamics of the fMRI signals from four brain regions. The strength of the non‐Gaussian innovations is controlled by the same parameter ψ. The non‐Gaussian innovations follow a t‐distribution with ν degrees of freedom and are identical for each brain region, which leads to fourth‐order connectivity as evidenced by coherent extreme fluctuations (i.e., both activations and deactivations) in the simulated fMRI signals (see Figure 1a) which is reflected in a positive cokurtosis and edge connectivity.

For later use, we briefly consider how the fourth‐order connectivity depends on the parameters ψ and ν. This may also provide some guidance to researchers using this model to generate synthetic fMRI signals with fourth‐order connectivity. Figure 2b, c show, respectively, the cokurtosis and edge connectivity, as a function of ψ for different values of ν. Note that, whereas their numerical values are different, the cokurtosis and edge connectivity depend on the model parameters in much the same way. In particular, for high values of ν, the innovations are nearly Gaussian and hence both measures are close to zero, whereas for small values of ν (and ψ=1), the innovations are strongly heavy‐tailed and hence both measures are large. Figure F and G show four simulated fMRI signals, respectively, without and with fourth‐order connectivity. It shows that the effects are subtle as well. However, in the presence of fourth‐order connectivity, we observe that the signals tend to simultaneously undergo extreme fluctuations.

#### 3.1.2 Bias, standard errors, and detection probabilities

In this section, we inspect the sampling distributions of the coskewness, cokurtosis, and edge connectivity estimators for resting‐state scanning sessions of 1200 samples and using a repetition time of 0.72 s. These are the settings of the resting‐state fMRI data of the HCP. We consider bias, standard errors, normality, and detection probabilities as a function of the model parameters. For all choices of the model parameters, the estimators' sampling distributions were approximated by generating a large number of synthetic fMRI datasets from the generative model and calculating the corresponding estimates. For the third‐ and fourth‐order estimators we generated, respectively, $10^{4}$ and $10^{5}$ synthetic datasets. For small values of ν, the sampling distribution of the cokurtosis and edge connectivity estimators have long right tails, which requires averaging over a larger number of datasets to be accurately represented. As in the previous simulations, we set τ=2 and r=0.4. We consider the sampling distributions as a function of ψ which is varied between 0 and 1. For the coskewness, α was varied between 0 and 3 and for the coskewness and edge connectivity ν ranged from 5 to 20 in steps of 3.

**FIGURE 3:**
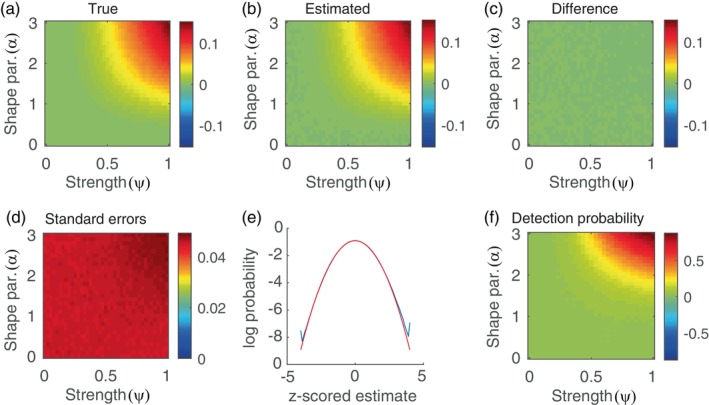
Sampling properties of the coskewness estimator. (a) True coskewness as a function of ψ and α. (b) The expected value of the coskewness estimator as a function of ψ and α, obtained from simulating $10^{4}$ synthetic functional magnetic resonance imaging (fMRI) data sets and averaging the estimates. (c) Bias of the coskewness estimator as a function of ψ and α. (d) Standard errors of the coskewness estimator as a function of ψ and α obtained by computing the sample standard‐deviation of the $10^{4}$ coskewness estimates. (e) Blue curve: Probability density of the z‐scored estimates, obtained by pooling the estimates for all values of ψ and α. Red curve: Standard normal density. (f) Detection probabilities as a function of ψ and α, obtained by z‐tests using a significance level of .05.

Figure 3a shows the true coskewness as a function of ψ and α and Figure 3b shows the expected value of the skewness estimator as a function of ψ and α. Their difference is shown in Figure 3c. It makes clear that the estimator is unbiased for resting‐state scanning sessions of this duration. Figure 3d shows the standard error of the estimator as a function of ψ and α and shows that it is more or less constant throughout the parameter space. Figure 3e (blue curve) shows the distribution of the coskewness estimater, which was obtained by pooling the z‐scored estimates of all parameter values (i.e., values of ψ and α). The red curve is the standard normal distribution and makes clear that the skewness estimator is normally distributed to a very good approximation.

These properties are relevant for practical applications, because they imply that the interval $\hat{\theta }\pm \sigma z_{\beta /2}$ is a $100\times \left(1-\beta \right)\%$ confidence interval for the coskewness, where $\hat{\theta }$ denotes the coskewness estimate, σ is the standard error of $\hat{\theta }$, and $z_{\beta }$ is the $100\times \left(1-\beta \right)\%$ percentile of the standard normal distribution. They also imply that $\hat{\theta }/\sigma$ is approximately standard‐normally distributed so that the null‐hypothesis $H_{0}:\theta =0$ can be tested using a z‐test. Figure 3f shows the probability of detecting the non‐zero coskewness as a function of ψ and α. It makes clear that the detection probability only exceeds its baseline level of 0.05 (the size of the test) if both ψ and α are large. The maximal detection probability is 87%, which is attained for ψ=1 and α=3.

**FIGURE 4:**
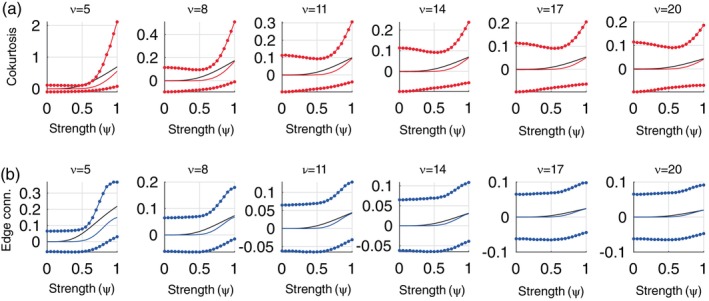
Sampling properties of the cokurtosis and edge connectivity estimators. Top row: True cokurtosis values (black curves), together with expected values of the plug‐in estimator (solid red curves) and the 2.5% and 97.5% percentiles of its sampling distribution (dotted red curves), as a function of ψ. The panels correspond to different values of ν. Bottom row: Same format but for the edge connectivity. Thus, for scanning sessions of this duration (14.4 min with a TR of 0.72) the fourth order estimators are generally negatively biased, which will lead to a loss of statistical power in hypothesis testing. Another property is that, except for ν=5 and large values of ψ, both the cokurtosis and edge connectivity estimators have very large uncertainties, which prevents the null hypothesis of no fourth‐order connectivity to be rejected. This suggests that they do not allow detection of fourth‐order connectivity in single‐subject data.

In contrast to the coskewness estimator, the cokurtosis and edge connectivity estimators systematically underestimate the true connectivities, except in the absence of higher‐order connectivity. This can be observed in Figure 4, which shows the true cokurtosis (panel A) and edge connectivity (panel B) values (black curves), together with the expected values of their estimators (red and blue curves, respectively). Figure 4 also shows the 2.5% and 97.5% percentiles of the estimators' sampling distributions (dotted lines). The intervals exclude the value zero only for ν=5 (and only for large values of ψ), which implies that if the estimators are used for testing the null hypothesis of no fourth‐order interaction, the detection probability will be zero for all values of ν except ν=5. We conclude that the statistical uncertainty in the estimators of fourth‐order connectivity is too large for them to be used for analyzing single‐subject resting‐state fMRI data, except when the signals strongly deviate from normality (i.e., when ν=5).

#### 3.1.3 Performance of the block bootstrap

Constructing confidence intervals or carrying out hypothesis tests for higher‐order connectivity in practice, requires an approximation of the estimators' sampling distribution. Although asymptotic results can be derived (see Appendices A and B), their derivation is based on the assumption of no auto‐correlations, which is generally invalid for fMRI signals, although the extent to which this assumption is violated will depend on how the signals are pre‐processed and in particular on the settings of the used band‐pass filter. Furthermore, although the null hypothesis of no higher‐order connectivity can in principle be tested by constructing null‐data, the authors are not aware of any method that yields null‐data for this particular hypothesis, unless the data are Gaussian, linear, and stationary, in which case coherent phase‐randomization can be used (Prichard & Theiler, 1994). In any case, null‐data cannot be used for constructing confidence intervals.

We thus explore the use of resampling methods, which can, in principle, be used for testing as well as for constructing confidence intervals for higher‐order connectivity. As described in Section 2.2.2, we focus on the block bootstrap (Hall et al., 1995; Kreiss & Paparoditis, 2011) which involves resampling (with replacement) blocks of consecutive observations, rather than individual observations, which is done to preserve the auto‐correlations in the signals. Indeed, sampling of individual observations, instead of blocks, will increase the probability of false rejections. To illustrate this, we generated synthetic fMRI data without third‐order connectivity and tested the null hypothesis of no third‐order connectivity, by resampling individual observations. All parameter values were the same as before (τ=2 samples, r=0.4, and N=1200 samples). Using a significance level of 0.05, the estimated fraction of rejected null hypotheses was 0.12±0.01.

We consider the performance of the block bootstrap as a function of scanning time, which ranged from 300 samples, which is one‐fourth of the scanning time of the HCP data, up to 4800 samples, which is four times the scanning time of the HCP data. The block‐size for the resampling was set to L=10 samples. This choice was based on the fact that the auto‐correlation function of the HCP data has a time‐scale of about two samples and hence reduces to zero in roughly 10 samples. In any case, experimenting with different block‐sizes made clear that the performance is not that sensitive to the block‐size as long as it is sufficiently large (results not shown). For each scanning time, we generated a synthetic fMRI data‐set without third‐order connectivity, computed the coskewness estimate and generated B=1000 bootstrap samples. From the bootstrapped samples, we estimated the estimators' standard error and used it to test the null‐hypothesis using a z‐test. This was repeated 1000 times, which allowed to estimate the bias in the bootstrap estimates of the standard‐error as well as the probability of rejecting the null hypothesis. The entire procedure was repeated 10 times to assess the level of uncertainty in the estimates of the bias and the rejection probabilities.

**FIGURE 5:**
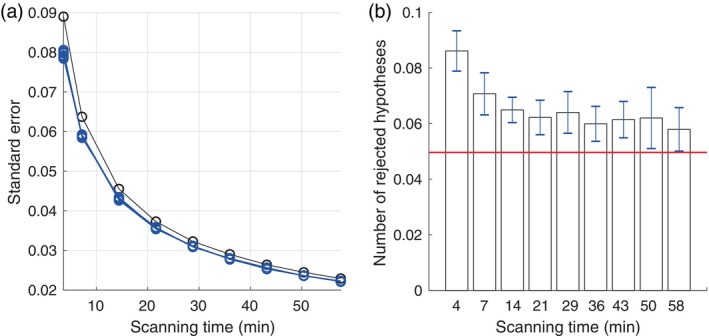
Performance of the block bootstrap. (a) The black circles correspond to the true standard errors of the coskewness estimator in the absence of third‐order connectivity (i.e., ψ=0) for the different scanning times. They were obtained by calculating $10^{5}$ coskewness estimates and taking their sample variance. The blue circles correspond to (estimates of) the bootstrap estimates of the standard errors. The small level of variability in the 10 blue lines shows that the estimates are rather precise. (b) Estimated probabilities of rejecting the null hypothesis (i.e., of making a type‐I error) for different scanning times. The error bars correspond to the standard errors of the probability estimates. The horizontal red line corresponds to the chosen significance level of .05. In panels (a) and (b), the scanning times included 300 samples and integer multiples of 600 samples up to 4800 samples. Repetition time was 0.72 s.

Figure 5a shows the true (black curve) and estimated (blue curve) standard errors as a function of scanning time. Note that the true standard errors decrease as the scanning time increases, reflecting the reduction in statistical uncertainty of the estimator when more data is available. The figure also shows that the block bootstrap systematically underestimates the standard errors, although the extent of which seems to decrease for longer scanning times. This implies that if the estimated standard errors are used for hypothesis testing, the probability of rejecting the null hypothesis will be higher than the used significance level. This can be observed in Figure 5b, which shows the estimated rejection rates for the different scanning times. The significance level was set at 0.05 and is designated by the horizontal red line. Although the rejection rates do decrease with increasing scanning time, they seem to stabilize at a value of around 0.06. Thus, scanning for longer than about 15 min does not substantially decrease the slightly inflated rejection rate of about 6%. Furthermore, for scanning sessions shorter than about 5 min, the rejection rate is substantially higher (about 9%). Based on these results, a tentative rule of thumb is to use scanning sessions of at least 10 min. However, the performance of the block bootstrap is likely to be sensitive to the exact settings of the used bandpass filter, since these directly affect the time‐scale of the auto‐correlations within the fMRI signals. The rule‐of‐thumb, however, also applies to fMRI signals with different repetition times, because what matters for performance is the effective number of samples, which is determined by the time‐scale of the auto‐correlations.

### 3.2 Empirical fMRI data

#### 3.2.1 Distributions of higher‐order connectivity

We estimated the cortical distributions of the plug‐in estimators of the coskewness, cokurtosis, and edge connectivity at the group‐level using the 94 subjects from the resting‐state HCP data (see Section 2.4 for details about the data‐set). For comparison, we also considered the distribution of the second‐order correlations. These distributions give an impression of the typical strength of higher‐order correlations in the data. We estimated the distributions by randomly selecting $10^{4}$ pairs/triplets/quadruplets of brain regions. For each connectivity measure, two random selections of $10^{4}$ pairs/triplets/quadruplets were made, to verify that the estimated distributions were independent of the selected regions and hence are accurate estimates of the distributions.

**FIGURE 6:**
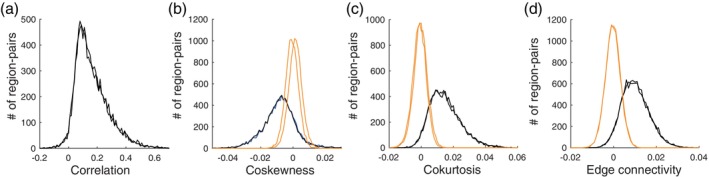
Group‐level distributions of higher‐order correlations. (a) Distribution of the second‐order correlations obtained by randomly selecting $10^{4}$ pairs of brain regions, calculating their sample correlation, and averaging over all 94 subjects. The two curves correspond to two independent selections of $10^{4}$ pairs of regions. (b) Black curves: Distribution of coskewness obtained by randomly selecting $10^{4}$ triplets of brain regions, calculating their sample coskewness, and averaging over all 94 subjects. The two black curves correspond to two independent selections of $10^{4}$ triplets of regions. Orange curves: Distribution of the coskewness obtained from phase‐randomized null‐data (two independent realizations). (c) Same format as (b) but for the cokurtosis. Instead of triplets, $10^{4}$ quadruplets were selected. (d) Same format as (b) but for the edge connectivity. Instead of triplets, $10^{4}$ quadruplets were selected.

Figure 6a shows the distributions of the second‐order correlations. The two curves correspond to the two independent selections of region‐pairs. They make clear that the distribution does not depend on the selected brain regions. Figure 6b (black curves) shows the distributions of the third‐order correlations (i.e., the coskewness). The values are about an order of magnitude smaller than those of the second‐order correlations. Specifically, for both selections, the average magnitude of the coskewness, relative to that of the correlations, is 6%. This value is similar to that reported in (Hlinka et al., 2011) for the relative magnitude of non‐Gaussian second‐order connectivity as measured by mutual information. This shows that, on the group‐level, third‐order correlations are relatively small compared to second‐order correlations. The estimates nevertheless are rather accurate: The average bootstrapped standard error is 0.006. Since the bootstrapped estimates are approximately normal (results not shown) this implies that absolute coskewness values larger than 1.96×standard error≈0.01 are significant at a significance level of *α* = .05 (uncorrected) which amounts to 38% of the selected triplets. For comparison, we also computed the distributions from coherent phase‐randomized surrogate data (orange curves in Figure 6b). In line with the above observation, they show that absolute values of the coskewness larger than about 0.01 cannot be explained by the Gaussian null hypothesis. Figure 6c shows that practically all significant coskewness values are negative.

Figure 6c shows that the cokurtosis values are also about an order of magnitude smaller than the second‐order correlations (average relative magnitude of 9% for both selections). The average bootstrapped standard error is 0.006, which amounts to significance of 59% of the selected quadruplets. Thus, the (uncorrected) threshold for statistical significance is about 0.01 which is in line with what is suggested by the null data (orange curves in Figure 6c). In contrast to the coskewness, all significant cokurtosis values are positive. The distribution of the coskewness is rather similar to that of the edge connectivity (compare with Figure 6d). Indeed, the Pearson correlation between the $10^{4}$ cokurtosis and the edge connectivity values is 0.93 (0.94 for the second selection) which shows that they practically capture the same features of the fMRI signals. The average bootstrapped standard error of the edge connectivity estimates is 0.004, which amounts to significance of 59% of the selected quadruplets.

#### 3.2.2 Higher‐order connectivity maps

In this section we inspect group‐level third‐order and fourth‐order connectivity maps for several seed regions and compare them to correlation maps. We start with the coskewness maps. Unlike correlation maps, which are constructed by fixing one seed region and calculating its correlation with a varying target region, a coskewness map is constructed by fixing *two seed regions* and calculating their coskewness with a varying target region. We thus selected four homologous pairs of seed regions in the primary motor cortex, frontal eye‐fields (which is part of the dorsal attention network), precuneus (which corresponds to the posterior medial node of the default mode network [DMN]), and the dorsolateral prefrontal node of the frontoparietal network. In the used cortical parcellation (Glasser, Coalson, et al., 2016), the selected regions in the precuneus and the dorsolateral prefrontal cortices are labeled “Area 31p ventral” and “Area 8C,” respectively. To compare the coskewness maps to correlation maps, the latter were averaged over the respective left and right seed regions.

**FIGURE 7:**
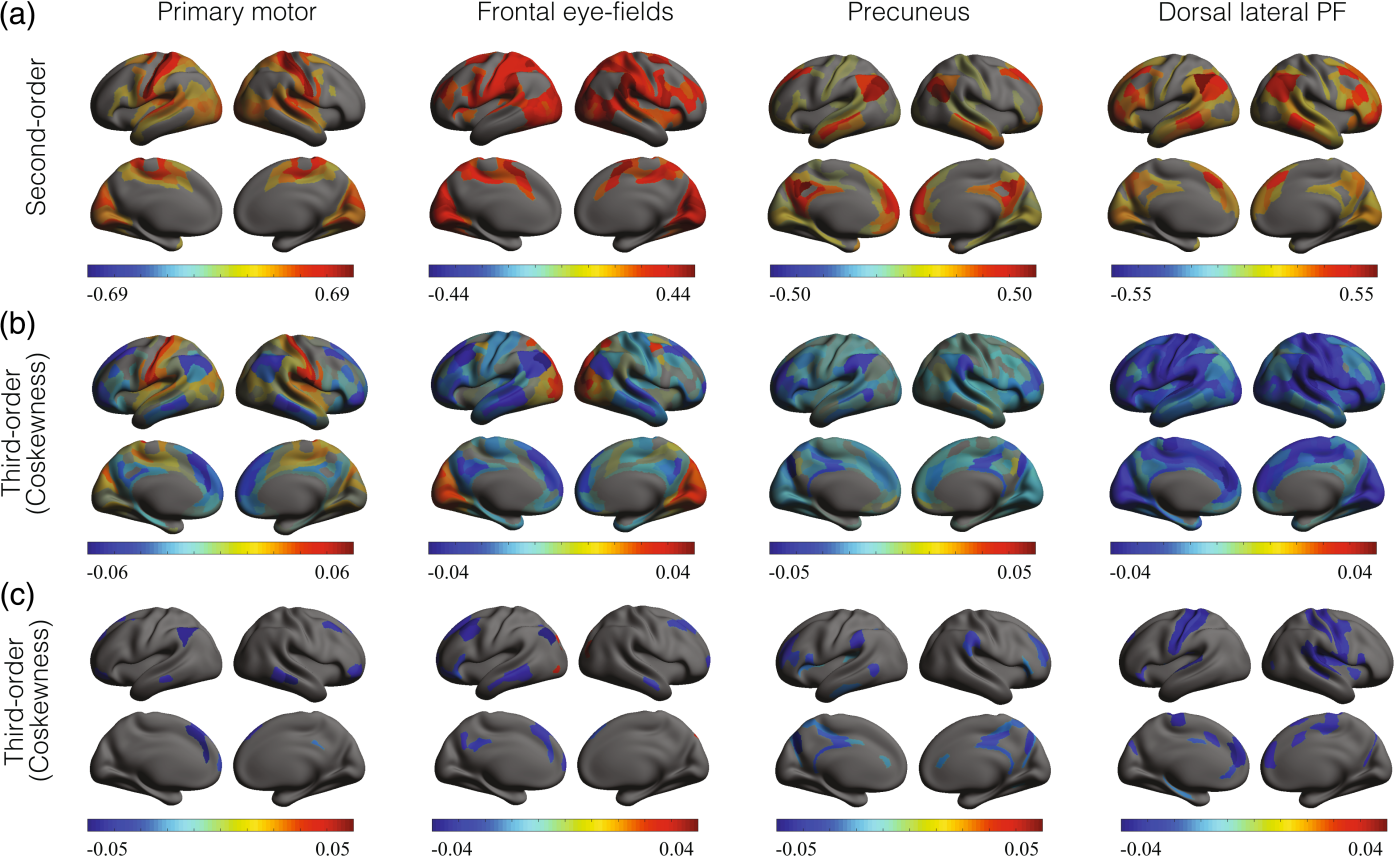
Third‐order correlation maps on the group‐level. (a) Group‐level second‐order correlation maps seeded in left and right primary motor cortex, frontal eye‐fields, precuneus, and dorsal lateral prefrontal cortex. The maps show the average second‐order correlation of all cortical regions with the left and right homologous seeds. The maps were thresholded at *p* < .01 (Bonferroni corrected) and subsequently at their median values (for better visualization). (b) Raw group‐level third‐order correlation (coskewness) maps for the same four pairs of homologous seeds as in (a). (c) Thresholded group‐level third‐order correlation (coskewness) maps for the same four pairs of homologous seeds at in Panel (a). The maps were thresholded at *p* < .01 (Bonferroni corrected).

Figure 7a shows the correlation maps for each of the four pairs of seed regions. The colors encode the average correlation of the fMRI signal in each cortical region with those in the respective pairs of seed regions. The maps were thresholded by bootstrapping the average of the left‐ and right‐seeded maps over subjects (*p* < .01, Bonferroni corrected). Thus, the colored regions are those for which the average correlation with the left and right primary motor cortices is significant at the chosen threshold. We recognize the motor network (seeds in primary motor cortex), the dorsal attention network (seeds in frontal eye‐fields), the DMN (seeds in precuneus), and the frontoparietal network (seeds in dorsal prefrontal cortex). Figure 7b, c show, respectively, the raw and thresholded (*p* < .01, Bonferroni corrected) coskewness maps, seeded in the same four pairs of regions. The raw maps are displayed to provide a more complete impression of their spatial structure, in addition to the rather conservatively thresholded maps shown in Figure 7c.

The coskewness maps differ from the correlation maps in two respects. First, they roughly comprise regions that are complementary to those that are correlated with the seeds regions. For example, whereas the fMRI signals in the primary motor cortices are predominantly correlated with those in the sensory cortices (somatosensory, auditory, and visual) (first panel in Figure 7a) the corresponding coskewness map shows the highest values in the regions that constitute the DMN (first panel in Figure 7c). For seeds in the frontal eye‐fields, the correlation map mostly covers posterior parietal and dorsal lateral frontal cortex (second panel of Figure 7a), whereas coskewness is high in regions that constitute the DMN (second panel of Figure 7c), which is roughly the complement of the dorsal attention network. Likewise, for seeds in the dorsal lateral prefrontal cortex, the regions with high coskewness, such as the motor cortex (third panel of Figure 7c), are complementary to those that constitute the frontoparietal network (third panel of Figure 7a). Finally, for seeds in the precuneus, whereas the correlation maps reflect the DMN (third panel of Figure 7a), the coskewness is high in the regions that constitute the frontoparietal network (third panel of Figure 7c).

A second difference between the correlation and coskewness maps is that, whereas the former is exclusively positive, the latter is (almost) exclusively negative, at least for the chosen significance level. As discussed in Section 2.1.1, there are two possible interpretations of a negative coskewness between three regions: either all three regions simultaneously undergo extreme deactivations or two of the three regions simultaneously undergo extreme activations, whereas the third region simultaneously undergoes extreme deactivations. We found that the first interpretation is correct in this case, which was checked by inspecting the signs of the correlations between the triplets of regions: all correlations were non‐negative. This shows that any three regions with significant coskewness engage in simultaneous extreme deactivations. Example fMRI signals with high coskewness are shown in figure 1 of Appendix C.

We next considered fourth‐order connectivity maps. We took the same four homologous region‐pairs as above and let two target regions vary over all homologous region‐pairs. The target regions were restricted to homologous region‐pairs to let the number of hypothesis tests be linear in the number of brain regions and to be able to visualize the maps on the cortex. In doing this, we implicitly assume that fourth‐order networks consist of homologous regions. Because this is true for most resting‐state networks, it is a reasonable initial assumption. In any case, it is straightforward to extend the analysis to non‐homologous regions, although the resulting maps are two‐dimensional (i.e., they are matrices) and hence cannot be displayed on the cortex.

**FIGURE 8:**
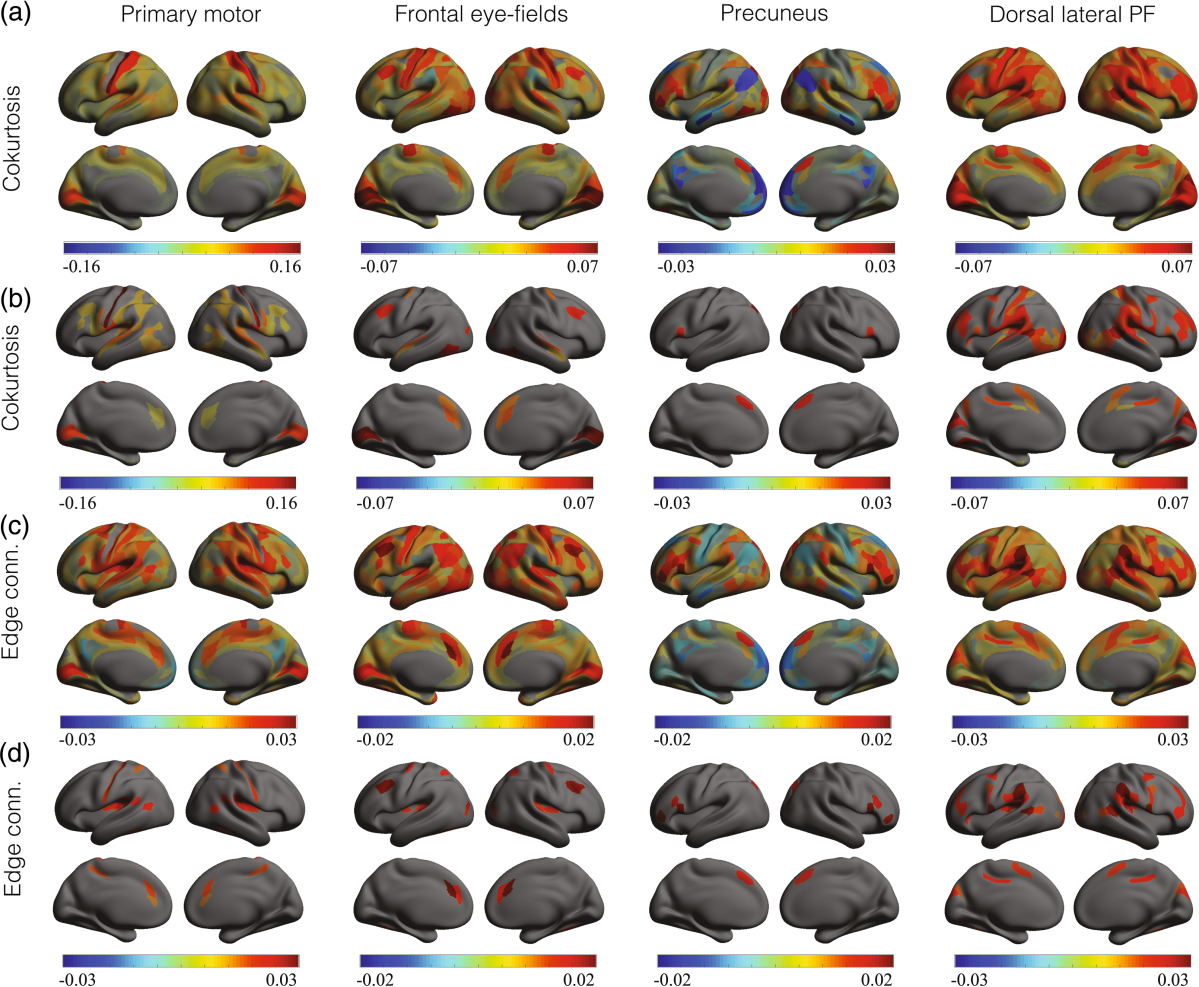
Fourth‐order correlation maps on the group‐level. (a) Raw group‐level cokurtosis maps seeded in left and right primary motor cortex, frontal eye‐fields, precuneus, and dorsal lateral prefrontal cortex. The maps show the cokurtosis of all homologous region‐pairs with the left and right homologous seed regions and hence are symmetric across hemispheres by construction. (b) Thresholded group‐level coskewness maps for the same four pairs of homologous seeds as in (a). The maps were thresholded at *p* < .01 (Bonferroni corrected). (c) Same format as (a) but for the edge connectivity. (d) Same format as (b) but for the edge connectivity.

Figure 8a, b show, respectively, the raw and thresholded (*p* < .01, Bonferroni corrected) cokurtosis maps for all four pairs of seed regions. In contrast to the coskewness maps, which were almost exclusively negative, the cokurtosis maps are exclusively positive, at least at the chosen significance level. This means that the fMRI signals in the four constituting regions of a quadruplet either undergo coherent fluctuations (i.e., both activations and deactivations) or split into two anti‐correlated pairs of coherent extreme fluctuations (see Figure 1a for an illustration). Because the pairwise correlations between the four regions were all non‐negative (results not shown) the fluctuations in all four regions are in fact coherent. Example fMRI signals with high cokurtosis are shown in figure 2 of Appendix C. Figure 8c, d show the raw and thresholded edge connectivity maps (*p* < .01, Bonferroni corrected) for all four pairs of seed regions. Although they differ from the cokurtosis maps in their details, overall they are very similar.

We also assessed if higher‐order connectivity can be detected in single‐subject data. As described in detail in Appendix D, this turns out to be challenging, even when omitting to correct for multiple testing. This finding is in line with our simulation results, which showed that detection probabilities of third‐order connectivity in single‐subject data are quite low, unless the signals are substantially non‐Gaussian (see Figure 3f) and that detection of fourth‐order connectivity from single‐subject data is practically impossible (see Figure 4). The reason for this, as suggested by our simulations, is the high sampling variability of the plug‐in estimators of the coskewness, cokurtosis, and edge‐connectivity.

#### 3.2.3 Clinical application (multiple sclerosis)

**FIGURE 9:**
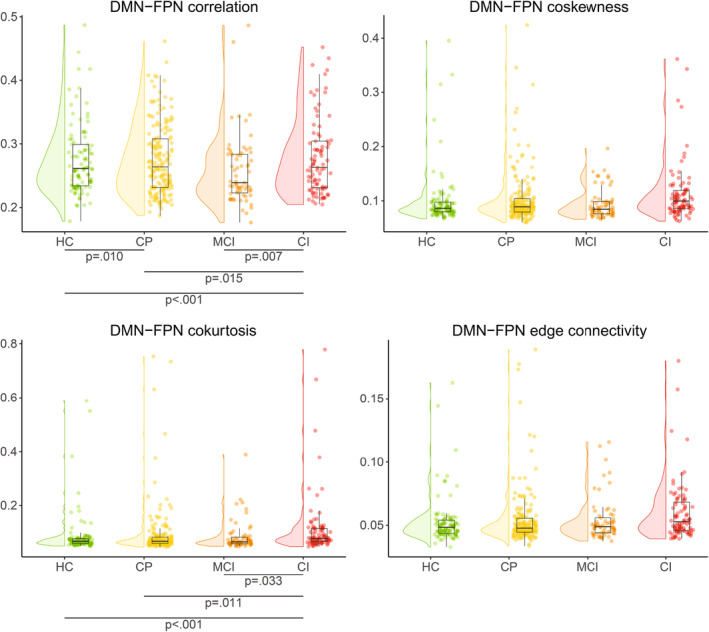
Higher‐order default‐mode network–frontoparietal network (DMN–fPN) connectivity in multiple sclerosis. DMN‐FPN pairwise connectivity and cokurtosis were increased in cognitively impaired (CI) patients compared to all other groups. Pairwise connectivity was additionally increased in preserved patients (CP) compared to healthy controls (HC). The colored points indicate the individual connectivity strengths that were observed for each individual and the distribution over all points is depicted to the left of them. These distributions clearly show non‐normality for the higher‐order measures, so these were log‐transformed in the statistical analyses.

No difference in within‐DMN second‐order correlations was observed between groups (*F*(3,416.8) = 0.76, *p* = .519, *p*‐adj = 1.000). However, dissimilar second‐order correlations between the FPN and DMN were observed (*F*(3,417) = 6.56, *p* < .001, *p*‐adj = .002), with the cognitively impaired group showing increased connectivity compared to all other groups (all *p* > .015) and cognitively preserved patients additionally showing increased second‐order correlations compared to healthy controls (*p* = .010). No other significant differences were observed (all *p* > .018 and *p*‐adj > .126). Therefore, higher‐order connectivity will be assessed between the DMN and FPN (see Figure 9).

Third‐order connectivity between the FPN and DMN can be defined using 2 nodes in the DMN and 1 in the FPN, or vice‐versa. We observed strong multicollinearity between these two combinations (VIF = 10.25), so we used the mean of both to characterize DMN‐FPN coskewness. This measure of mean DMN‐FPN coskewness did not differ between groups (*F*(3,417) = 1.94, *p* = .123).

Fourth‐order connectivity can be defined using three nodes in the DMN and one in the FPN, three in the FPN and one in the DMN, and by two nodes in both networks. We observed high multicollinearity for cokurtosis (VIF > 18.07) as well as edge connectivity (VIF > 13.56), so mean values across all three combinations were used. Mean DMN‐FPN cokurtosis was different between groups (F(3,417) = 4.30, *p* = .005), with CI (cognitively impaired) showing increased DMN‐FPN cokurtosis compared to all other groups (all *p* < .033). Mean DMN‐FPN edge connectivity was not different between groups (F(3,417) = 2.27, *p* = .080).

A multiple linear regression model was used to predict average cognition scores, with the following independent variables being added in consecutive steps: (1) age, sex and education, (2) pairwise global and DMN‐FPN connectivity, and finally (3) global and DMN‐FPN cokurtosis (see Table 1). In the final model, patients' average cognition was significantly predicted (adj. $R^{2}$ = 0.146, F(7,322) = 9.01, *p* < .001) using the following variables: age (β = −.194, *p* < .001), sex (β = −.162, *p* = .003), education (β = .220, *p* < .001) and DMN‐FPN cokurtosis (β = −.217, *p* = .004). The change in explained variance from step 1 (adj. $R^{2}$ = 0.120) to step 2 (adj. $R^{2}$ = 0.121) did not increase significantly ($R^{2}$‐change = 0.01, *F*(2,324) = 1.02, *p* = .364). However, the explained variance did increase from step 2 to step 3 ($R^{2}$‐change = 0.03, *F*(2,322) = 5.76, *p* = .003).Further, also adding all other pairwise connectivity values in the second step did not affect the outcome, as none of those values significantly predicted cognition (all *p* < .191) whereas DMN‐FPN cokurtosis still did (β = −.209, *p* = .007) and the explained variance still increased from step 2 to step 3 ($R^{2}$‐change = 0.03, F(2,316) = 5.03, *p* = .007).

**TABLE 1:** Fourth‐order DMN‐FPN cokurtosis explained significant additional variance in the average cognition of MS patients.

Models variables	Step 1: Clinical variables β (*p*‐value)	Step 2: +Correlation *β* (*p*‐value)	Step 3: +Cokurtosis *β* (*p*‐value)
Age	**−.211 (<.001)**	**−.199 (<.001)**	**−.194 (<.001)**
Sex	**−.135 (.010)**	**−.157 (.004)**	**−.162 (.003)**
Education	**.235 (<.001)**	**.230 (<.001)**	**.235 (<.001)**
Global correlation		.121 (.171)	.142 (.108)
DMN‐FPN correlation		−.071 (.406)	−.027 (.748)
Global cokurtosis			.052 (.484)
DMN‐FPN cokurtosis			**−.217 (.004)**
	**Adj. *R*** ^**2**^ = **0.120**	Adj. *R* ^2^ = 0.121 *p*‐change = .364	**Adj. *R*** ^**2**^ **= 0.146** ***p*‐change = .003**

Abbreviations: DMN–fPN, default‐mode network–frontoparietal network; MS, multiple sclerosis. Variables that added significantly to the model (i.e., *p*‐value <.05) were highlighted by making them bold. Similarly, models that explained additional variance beyond the previous step were made bold as well.

## 4 DISCUSSION

### 4.1 Summary and significance

In this study we proposed measures for quantifying genuine higher‐order connectivity in fMRI data, analyzed the performance of plug‐in estimators using a non‐Gaussian generative model of fMRI signals, compared two methods for statistical inference, and applied the methodology to two resting‐state fMRI datasets. The main advantage of using multivariate cumulants over the currently used edge connectivity (Faskowitz et al., 2020) is that multivariate cumulants vanish if higher‐order connectivity can be reduced to pairwise connections (i.e., is redundant), whereas this is not the case for the edge connectivity (Novelli & Razi, 2022). With respect to the simulations, our main conclusions are that, whereas third‐order connectivity can be detected in single‐subject data from single scanning sessions, fourth‐order connectivity can only be detected if it is unrealistically strong. Furthermore, whereas for scanning sessions of about 5 min, block bootstrapping leads to inflated false‐positive rates (9% at *α* = .05), for sessions of 10 min or longer, the errors rates are only slightly inflated (6% at *α* = .05). With respect to the application to fMRI data, we demonstrated the existence of third‐ and fourth‐order functional networks on the group‐level that are complementary to known (second‐order) resting‐state networks. Finally, we observed that higher‐order connectivity, especially based on fourth‐order cumulants, was stronger in patients with MS who show cognitive impairment. Higher‐order connectivity explained additional variance beyond pairwise connectivity, hence showcasing the relevance of non‐redundant higher‐order connections in the study of clinical samples.

### 4.2 Results on the HCP data‐set

Concerning the group‐level analysis, we estimated the cortical distributions of third‐ and fourth‐order correlations and compared them with that of the second‐order correlation. The values of the third‐ and fourth‐order correlations were comparable with each other and about an order of magnitude smaller than the values of the second‐order correlation. This observation is in line with an earlier study (Hlinka et al., 2011) in which the mutual information and its Gaussian part were compared on resting‐state fMRI data. The authors of (Hlinka et al., 2011) found that the portion of the mutual information that was not captured by its Gaussian part was rather small (about 5%) and concluded that the Gaussian part is sufficient for analyzing (pairwise) functional connectivity. Whereas the focus of (Hlinka et al., 2011) was on the effects of non‐Gaussianity on conventional functional connectivity analysis, the focus of our study is on the deviations from Gaussianity themselves and their cortical organization. From this point of view, the following observations are of interest.

First, all significant third‐order correlations were negative. In Section 3.2.2, we established that this reflects the fact that the fMRI signals of all three brain regions undergo simultaneous extreme (i.e., non‐Gaussian) deactivations. This implies that the signals themselves are (negatively) skewed, since symmetric signals cannot have non‐zero moments (see Appendix E). In other words, deactivations are more extreme than activations. Furthermore, the correlation between these extreme deactivations cannot be explained by the pairwise correlations between the three pairs of regions that constitute the triplet. In this respect, the triplet should be regarded as an integrated functional unit that cannot be decomposed. Concerning the fourth‐order correlations, all significant cokurtosis and edge connectivity values were positive and we established that this reflects the fact that the fMRI signals of all four brain regions undergo simultaneous extreme fluctuations that cannot be explained by the pairwise correlations between the six constituting pairs of regions. In this respect, the quadruple should also be regarded as an integrated functional unit that cannot be decomposed.

We also explored the spatial organization of group‐level third‐ and fourth‐order correlations by using appropriate seed‐based correlation maps. Third‐order correlation maps were constructed by fixing two (homologous) reference regions and varying a third target region over the cortex. We considered four pairs of seed regions, each of which is part of a well‐known resting‐state network. The first finding is that, for each of the four pairs of seed regions, there are several cortical areas that have significant third‐order correlations with the seed regions. These regions thus survived the tight statistical threshold of *α* = .01 with Bonferroni correction for conducting 360 hypothesis tests corresponding to the 360 target regions. Similar observations apply to the fourth‐order correlations. We therefore conclude that, although higher‐order correlations in resting‐state fMRI data are rather weak, they are a robust feature of the data, at least on the group‐level.

A second finding is that the regions with significant coskewness with the seed regions, roughly form the complement of the regions with significant second‐order correlation with the seed regions. This was observed for all four pairs of seed regions. For example, for seeds in the primary motor cortices, regions with significant second‐order correlation included premotor and sensory (somatosensory, visual, and auditory) regions, whereas the regions with significant third‐order correlation only included association regions (default mode regions). This observation demonstrates that coskewness maps capture aspects of the functional organization of spontaneous cortical fluctuations that are distinct and complementary to those provided by second‐order correlation maps.

### 4.3 Results on the MS data‐set

Concerning the analysis of the MS dataset, we observed increased pairwise (consistent with previous analyses (Meijer et al., 2017)) and cokurtosis between the DMN and FPN in cognitively impaired patients compared to all other groups. No differences between groups were observed for higher‐order connectivity metrics investigated in this work (coskewness and edge connectivity). The specific effects of DMN‐FPN cokurtosis and not of any other higher‐order metric are interesting and highlight the importance of looking at these orders individually, which is consistent with previous observations using higher‐order connectivity based on information theory (Herzog et al., 2022). With respect to third‐ versus fourth‐order connectivity, one important difference is the sensitivity of third‐order connectivity to deactivations which might be less relevant to cognitive impairment. Deactivations might, in theory, put less computational pressure on the network compared to coactivations and thereby might not contribute to an overload of brain hubs (Schoonheim et al., 2022). Although DMN‐FPN edge connectivity was not different between groups, the effect did show comparable directionality to DMN‐FPN cokurtosis, which is unsurprising given the strong overlap between the two measures. The slightly heightened sensitivity of cokurtosis might be due to the fact that edge connectivity depends on the (common) correlation between the edge signals from different brain regions, which may also include pairwise (Gaussian) terms, in contrast to cokurtosis, where only statistically genuine fourth‐order connectivity is taken into account. Importantly, DMN‐FPN cokurtosis explained additional variance beyond pairwise connectivity, which further emphasizes the clinical relevance of higher‐order connectivity. For MS specifically, these results reiterate that functional reorganization might play an important role in cognitive impairment (Fleischer et al., 2019; Schoonheim et al., 2022). Nevertheless, to be sure that higher‐order connectivity captures functional reorganization in MS, more work is needed that investigates the interaction between increased higher‐order connectivity and structural disconnections. Additionally, future studies could try to disentangle how the different orders of connectivity relate to distinct biological processes, as this could help interpret why we only observed differences in DMN‐FPN cokurtosis (fourth‐order connectivity).

### 4.4 Higher‐order connectivity in single‐subject data

Although robust higher‐order networks could be extracted at the group‐level, it was considerably more difficult to extract them from single‐subject data, due to the large variance of the plug‐in estimators (see Appendix D). A possible way to deal with this is to consider parametric estimators, since these usually have smaller variance than non‐parametric estimators. This requires selecting a parametric family of probability distributions that generalize the multivariate normal distribution and that allow multivariate cumulants to be calculated in closed‐form in terms of the model parameters. Third‐order connectivity requires a generalization of the three‐dimensional normal distribution that allows for asymmetry and hence can model data with non‐zero coskewness. Fourth‐order connectivity requires a generalization of the four‐dimensional normal distribution that allows for heavy or light tails and hence can model data with non‐zero cokurtosis. Families of probability distributions with these properties are the generalized skew‐elliptical (Branco & Dey, 2001) and elliptical distributions (Cambanis et al., 1981). However, it is unclear if these distributions can adequately model triplets and quadruplets of resting‐state fMRI signals and how accurately their parameters can be estimated from single‐subject data.

### 4.5 Cokurtosis versus edge connectivity

In this study, we considered two fourth‐order connectivity measures, namely, the cokurtosis and the recently proposed edge connectivity (Faskowitz et al., 2020). As pointed out in (Novelli & Razi, 2022), edge connectivity cannot directly be used as a measure for connectivity between edges, because it might reflect pairwise connectivity between the four regions that constitute the edges. In this sense, it is redundant and needs to be adjusted by subtracting its redundant (i.e., Gaussian) part. Which of the two measures, cokurtosis or the adjusted edge connectivity, should be preferred? In our generative model of fMRI signals, the cokurtosis and adjusted edge connectivity depend in a similar way on the model parameters and, in this sense, measure the same quantity. Furthermore, their plug‐in estimators behave comparably, both in simulated and empirical fMRI data, although their connectivity maps differ in some details. In the clinical application, however, the cokurtosis, but not the adjusted edge connectivity, picked up a difference between groups. Although one of the advantages of edge connectivity, as defined in (Faskowitz et al., 2020), is that it ranges between −1 and 1, this is not true anymore for its adjusted version. From a practical perspective, therefore, neither of the measures is to be preferred over the other. From a theoretical perspective, the cokurtosis is perhaps preferable, because it is simpler and can directly be generalized to orders higher than four. A more complete answer to this question will likely depend on the results of future fMRI studies.

### 4.6 Do fMRI signals have finite moments?

A potential issue is that the multivariate moments, in terms of which the higher‐order connectivity measures are defined, are infinite. This issue, however, is not particular to higher‐order multivariate moments, but also applies to first‐ and second‐order univariate moments. For example, if resting‐state fMRI signals follow a t‐distribution with two degrees of freedom, their variance is infinite and this would complicate any statistical inference that involves the signals' variances. Although it is generally not possible to decide, based on signals of finite length, if the signals' moments are finite or infinite, an indication can be obtained by plotting the sample moments as a function of the sample size (Granger & Orr, 1972). Because the sample moments are consistent and their sampling variability approaches zero when the sample size becomes large (see Appendices A and B), the finiteness of the signals' theoretical moments implies that its sample moments converge to finite values. In Appendix F, we plot the first four moments of a large number of resting‐state fMRI signals. The moments indeed seem to converge to finite values, which provides some evidence that the theoretical moments up to order four are indeed finite. A related issue is that the moment generating function of multivariate fMRI signals might not exist. In particular, the finiteness of the signals' moments is not a sufficient condition for the existence of its moment generating function. However, the moment generating function was only used in this study to introduce higher‐order moments and cumulants in a unified way and is in no way essential. The only requirement for the higher‐order connectivity measures to be well‐defined is that the moments up to order four are finite.

### 4.7 Connections to information theory and topological data analysis

This work relates to two other higher‐order interaction approaches: information theory and topological data analysis (TDA). In information theory, synergistic interactions are defined as interactions that only exist in high order but not necessarily in pairwise. In contrast, our work investigates higher‐order interactions as measured by multivariate cumulants. Our results suggest that higher‐order connectivity is related to the DMN, which is consistent with recent developments in information theory for neuroscience (Luppi, Mediano, Rosas, Holland, Fryer, O'Brien, et al., 2022; Santos et al., 2023). Considering the similarities between multiple approaches for quantifying higher‐order interactions, exploring their relationships in future research would be valuable. Some key issues that warrant investigation include comparing the sensitivity and specificity of information‐theoretic measures and multivariate cumulants in detecting higher‐order interactions in fMRI data, assessing the extent to which synergistic interactions identified through information‐theoretic approaches overlap with those identified using multivariate cumulants, and investigating the potential for combining statistical and information‐theoretic approaches to develop a unified framework for characterizing higher‐order connectivity in the brain. Since any random variable (with finite moments) can be expressed in terms of cumulants, this could provide a foundation for connecting statistical and information‐theoretic approaches to higher‐order interactions in complex systems. Establishing such connections may lead to a more comprehensive understanding of higher‐order interactions in fMRI data and may offer new avenues for investigating neuropsychiatric diseases and cognitive neuroscientific experiments.

The application of higher‐order cumulants to TDA represents a potential follow‐up of our work. Central to TDA is the construction of simplicial complexes from the observed data and comprise nodes, edges, triangles, tetrahedral, and their higher‐order versions. Simplicial complexes are typically derived from pairwise connectivities and identified by clique computations (Centeno et al., 2022). However, it can be argued that the basis for these structures could be substantially improved by utilizing simplicial complexes derived from high‐order cumulants that exclude redundant second‐order correlations. We believe that this may not only refine our understanding of simplicial complexes, but also offer insight into other complex structures like hypergraphs. Therefore, the potential of higher‐order cumulants in developing more accurately informed simplicial complexes and hypergraphs deserves further investigation.

A related issue is the distinction between statistical and topological higher‐order connectivity. The first, the central focus of this work, is concerned with whether there exist statistical dependencies between more than two variables, that cannot be reduced to dependencies between pairs of variables. However, an interaction can be topologically higher‐order, even when the underlying mechanisms are pairwise (Rosas et al., 2022). For instance, higher‐order effects can be observed in a pairwise computational model of whole‐brain dynamics (Gatica et al., 2022). In this sense, measures that are based on pairwise connectivity, such as cliques, cluster coefficients, communities or modularity (Centeno et al., 2022), can yield different results if one defines the weights of the higher‐order structures via higher‐order moments, regardless of their statistical origin. Finally, in our study, we focused on higher‐order connectivity at the level of individual triples/quadruplets, and did not consider the topological structure of higher‐order networks, as characterized by simplicial complexes or hypergraphs (Bick et al., 2022). Our results indicate that coskewness and cokurtosis could define reliable weights for constructing hyperedges and simplicial complexes.

## AUTHOR CONTRIBUTIONS

Contribution of all authors is listed here: study design/conceptualization (RH, MMS, LD, PKBT), data analysis/formal analysis (RH, TAAB), methodology/development method pipeline (RH, WvW), software (RH), data interpretation (PKBT, RH, TAAB, MMS, LD, FS, WvW), data curation (RH, TAAB, MMS), visualization (RH, TAAB), writing manuscript (RH, TAAB, FS, PKBT), editing manuscript (RH, TAAB, MMS, LD, FS, WvW, PKBT).

## CONFLICT OF INTEREST STATEMENT

The authors declare no conflicts of interest.

## Supporting information


**Data S1.** Supporting information.

## Data Availability

The data that support the findings of this study are available in Human Connectome Project at https://db.humanconnectome.org/app/template/Login.vm. These data were derived from the following resources available in the public domain: HCP, https://db.humanconnectome.org.
