# Supplementary material for: Higher‐order functional connectivity analysis of resting‐state functional magnetic resonance imaging data using multivariate cumulants
Source: Hum Brain Mapp. 2024 Mar 23;45(5):e26663. doi: 10.1002/hbm.26663 (PMC10960559; doi:10.1002/hbm.26663)
Supplement: Supplementary file 1 — Data S1. Supporting information. [file HBM-45-e26663-s001.pdf]

Appendices to: Higher-order functional connectivity analysis of resting-state fMRI data using multivariate cumulants

Rikkert Hindriks[1], Tommy A.A. Broeders[2], Menno M. Schoonheim[2], Linda Douw[2], Fernando Santos[3,4], Wessel van Wieringen[5], and Prejaas K.B. Tewarie[6,7]

[1]Department of Mathematics, Faculty of Science, Vrije Universiteit Amsterdam, Amsterdam, The Netherlands

[2]Department of Anatomy and Neurosciences, Amsterdam Neuroscience, Amsterdam UMC, Vrije Universiteit Amsterdam, Amsterdam, The Netherlands

[3]Dutch Institute for Emergent Phenomena (DIEP), Institute for Advanced Studies, University of Amsterdam, Amsterdam, The Netherlands

[4]Korteweg de Vries Institute for Mathematics, University of Amsterdam, Amsterdam, the Netherlands

[5]Department of Epidemiology and Biostatistics, Amsterdam UMC, Vrije Universiteit Amsterdam, Amsterdam, The Netherlands

[6]Clinical Neurophysiology Group, University of Twente, Enschede, The Netherlands

[7]Sir Peter Mansfield Imaging Center, School of Physics, University of Nottingham, Nottingham, United Kingdom

December 22, 2023

A. Asymptotic sampling distributions of coskewness and cokurtosis

Let $X \in \mathbb{R}^n$ be a zero-mean random vector modeling the fMRI signals from n brain regions. The coskewness between regions i, j, k is defined as

$$r^c_{i,j,k} = \frac{\mathbb{E}[X_i X_j X_k]}{\left(\mathbb{E}[X_i^2]\mathbb{E}[X_j^2]\mathbb{E}[X_k^2]\right)^{3/2}},$$

and its plug-in estimator is obtained by replacing the expectations by sample averages over a random sample of size N:

$$\hat{r}^c_{i,j,k} = \frac{\langle X_i X_j X_k \rangle}{\left(\langle X_i^2 \rangle \langle X_j^2 \rangle \langle X_k^2 \rangle\right)^{3/2}}.$$

We derive the asymptotic distribution of $\hat{r}^c_{i,j,k}$. Define the random vector $Y \in \mathbb{R}^4$ by

$$Y = (X_i X_j X_k, X_i^2, X_j^2, X_k^2),$$

and let θ and Ω be, respectively, the expected value and covariance matrix of Y. Application of the central limit theorem to the sample mean $\langle Y \rangle$ shows that $\sqrt{N}(\langle Y \rangle - \theta) \sim N(0, \Omega)$ for large N. We now apply Cramer's theorem to the function

$$g(x, y, z, u) = \frac{x}{(yzu)^{3/2}},$$

which shows that $\sqrt{N}(g(\langle Y \rangle) - g(\theta)) \sim N(0, \nabla_g(\theta)'\Omega\nabla_g(\theta))$, where $\nabla_g(\theta)$ is the gradient of g at θ. But

$$\theta = \left(\mathbb{E}[X_i X_j X_k], \mathbb{E}[X_i^2], \mathbb{E}[X_j^2], \mathbb{E}[X_k^2]\right),$$

and therefore $g(\theta) = r^c_{i,j,k,}$. Furthermore, $g(\langle Y \rangle) = \hat{r}^c_{i,j,k}$ so that $\sqrt{N}(\hat{r}^c_{i,j,k} - r_{i,j,k}) \sim N(0, \nabla_g(\theta)'\Omega\nabla_g(\theta))$ for large N. This shows that $\hat{r}^c_{i,j,k}$ is a consistent estimator of $r^c_{i,j,k}$ and is asymptotically normal with variance $\sigma^2_{\hat{r}} = \nabla_g(\theta)'\Omega\nabla_g(\theta)/N$.

B. Asymptotic sampling distribution of the edge connectivity

Let $X \in \mathbb{R}^n$ be a zero-mean random vector and let $f_1(X)$ and $f_2(X)$ be monomials in the components of X. Below we suppress the dependence of f_1 and f_2 on X. The edge connectivity has the following form:

$$r = \frac{\mathbb{E}[f_1 f_2]}{\sqrt{\mathbb{E}[f_1^2]\mathbb{E}[f_2^2]}},$$

for particular choices of f_1 and f_2, and its plug-in estimator is defined by replacing expectations by sample averages over a random sample of size N:

$$\hat{r} = \frac{\langle f_1 f_2 \rangle}{\sqrt{\langle f_1^2 \rangle \langle f_2^2 \rangle}}.$$

We derive the distribution of $\hat{r}$ for large N. Define the random vector $Y \in \mathbb{R}^3$ by

$$Y = (f_1 f_2, f_1^2, f_2^2)'$$

where $'$ denotes the transpose, and let θ and Ω be, respectively, the expected vector and covariance matrix of Y. Application of the central limit theorem to the sample mean $\langle Y \rangle$ shows that $\sqrt{N}(\langle Y \rangle - \theta) \sim N(0, \Omega)$ for large N. We now apply Cramer's theorem to the function $g(x, y, z) = x/\sqrt{yz}$, which shows that $\sqrt{N}(g(\langle Y \rangle) - g(\theta)) \sim N(0, \nabla_g(\theta)'\Omega\nabla_g(\theta))$, where $\nabla_g(\theta)$ is the gradient of g at θ. But

$$\theta = \left(\mathbb{E}[f_1 f_2], \mathbb{E}[f_1^2], \mathbb{E}[f_2^2]\right)'$$

hence $g(\theta) = r$, and furthermore, $g(\langle Y \rangle) = \hat{r}$, so that $\sqrt{N}(\hat{r} - r) \sim N(0, \nabla_g(\theta)'\Omega\nabla_g(\theta))$ for large N, where

$$\nabla_g(\theta) = \left(\frac{1}{\sqrt{\mathbb{E}[f_1^2]\mathbb{E}[f_2^2]}}, -\frac{r}{2\mathbb{E}[f_2^2]}, -\frac{r}{2\mathbb{E}[f_1^2]} \right)'.$$

This shows that $\hat{r}$ is a consistent estimator of r and is asymptotically normal with variance $\sigma_{\hat{r}}^2 = \nabla_g(\theta)'\Omega\nabla_g(\theta)/N$.

C. Example fMRI signals with strong higher-order interactions

We selected the three subjects with the largest absolute average coskewness with the primary motor cortices and subsequently selected the fMRI signals from the primary motor cortices and from the target region with the largest absolute coskewness. The signals are shown in Figure 1. We indeed observe coherent extreme deactivations of all three regions are several moments of the scanning session, for example at around 200 seconds in HCP subject 9.

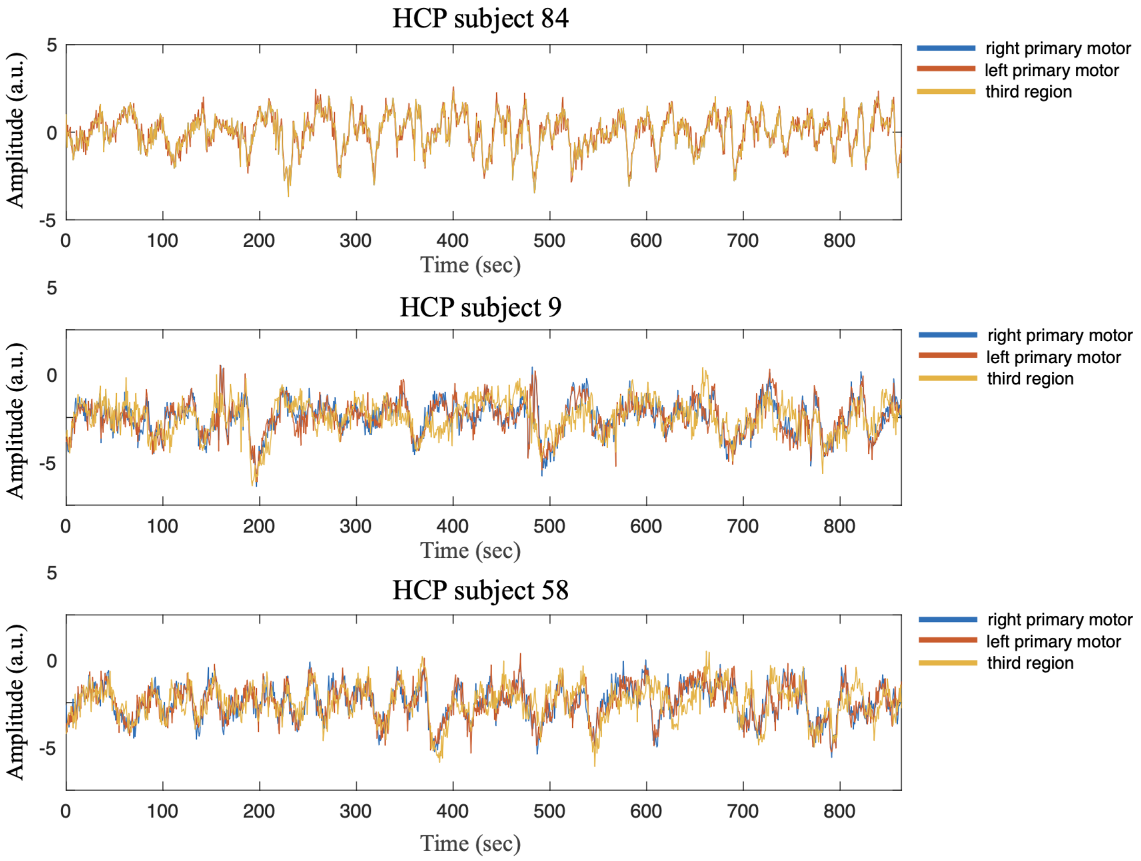

Figure 1: *fMRI signals with strong third-order correlation.* fMRI signals from the left (red) and right (blue) primary motor cortex, and a target cortical region (yellow) for three different HCP subjects. For each of the three subjects, the target region was chosen so that its average third-order correlation with the fMRI signals in the primary motor cortices was maximal in absolute value. For better visibility, all fMRI signals were normalized to unit variance.

We selected the three subjects with the largest absolute average cokurtosis with the primary motor cortices and subsequently selected the fMRI signals from the primary motor cortices and from the two target regions with the largest absolute coskewness. The signals are shown in Figure 2. We indeed observe coherent extreme fluctuations of all four regions are several moments, for example at around 200 seconds in HCP subject 9.

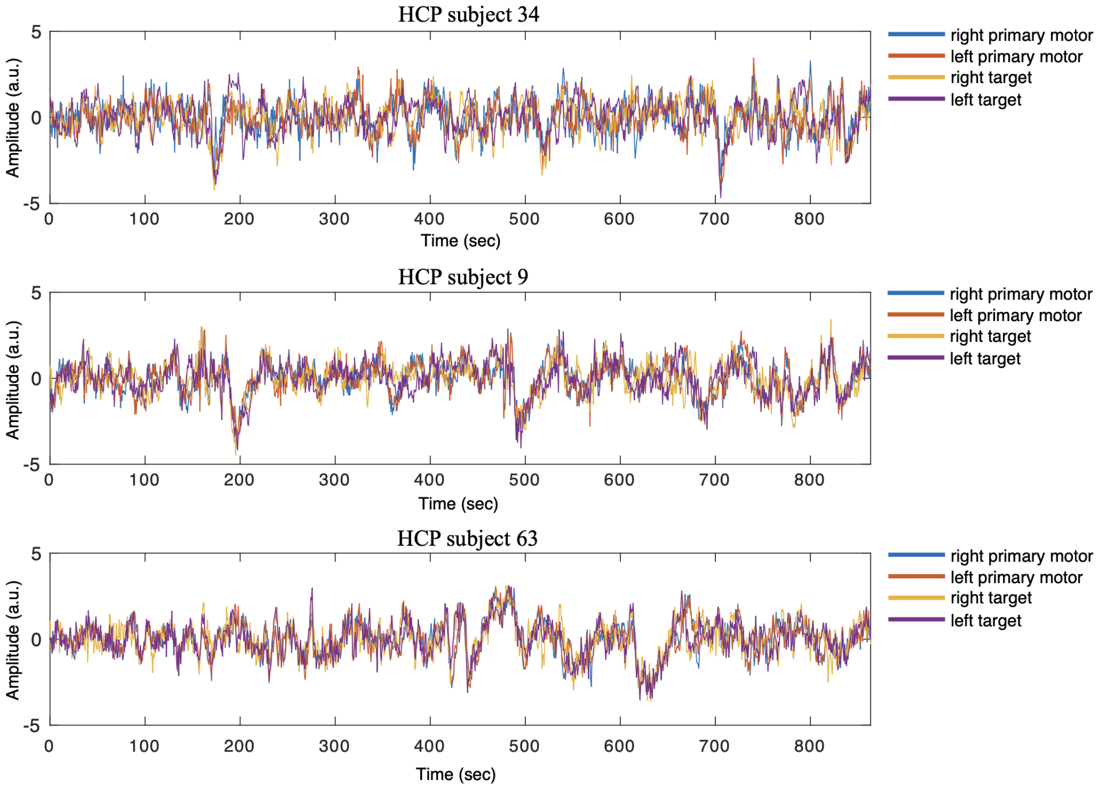

Figure 2: *fMRI signals with strong fourth-order correlation.* fMRI signals from the right (blue) and left (red) primary motor cortex, together with right (yellow) and left (purple) target regions for three different HCP subjects. For each of the three subjects, the target regions were chosen so that their average fourth-order correlation with the fMRI signals in the primary motor cortices was maximal in absolute value. For better visibility, all fMRI signals were normalized to unit variance.

D. Higher-order connectivity maps in single-subject data

To assess if higher-order connectivity can be detected in single-subject data, we used one of the four homologous region-pairs from Section 3.2.2 in the main text, namely the frontal eye-fields. As in Section 3.2.2 of the main text, we tested for statistical significance of third-order connectivity with all cortical regions and for fourth-order connectivity with all homologous region-pairs. The resulting seed-based maps can therefore directly be compared to those obtained on the group-level. Testing was done using the block bootstrap with block-length $L = 10$ samples by computing 95% confidence intervals, based on 1000 bootstrap realizations. In contrast to the simulated data, the connectivity estimates were not entirely normally distributed, so that a Gaussian approximation to the sampling distributions could not be used to obtain the intervals. The intervals were therefore computed by taking the 2.5% and 97.5% percentiles of the sampling distributions.

The raw seed-based maps are displayed in Figure 1A (coskewness), B (cokurtosis) and C (edge connectivity). Note the large inter-subject variability in the maps. Specifically, the average spatial correlation between the maps from different subjects is 0.08 ± 0.24 (coskewnss), 0.02 ± 0.19 (cokurtosis), and 0.01 ± 0.14 (edge connectivity). The simulations carried out in Section 3.1.2 of the main text suggest that this variability reflects statistical uncertainty, rather than variability in the true connectivity maps. This is also suggested by the numerical values of the connectivity estimates (see the colorbars in Figure 1A-C), which are about an order of magnitude larger than those obtained on the group-level (see Figure 6 of the main text). To confirm this, we computed the spatial correlations of the maps with those obtained from a separate scanning session. The subject-averaged spatial correlations were 0.17 ± 0.30 (coskewness), 0.10 ± 0.23 (cokurtosis), and 0.08 ± 0.17 (edge connectivity). These low values show that the intra-subject variability in the connectivity maps is particularly large. For comparison, the subject-averaged spatial correlation between the second-order correlations maps obtained from two separate scanning sessions is 0.66 ± 0.12. From this analysis we therefore conclude that the large inter-subject variability in the third- and fourth-order connectivity maps indeed reflects statistical uncertainty.

The thresholded seed-based maps are shown in Figure 1D (coskewness), E (cokurtosis), and F (edge connectivity). The figures show, for each cortical region, the number of subjects (out of a total of 94 subjects) for which the fMRI signal from that region was significantly connected to the two reference regions (left and right frontal eye-fields). The maps resemble the corresponding group-level maps in Figures 7 (coskewness) and 9 (cokurtosis and edge connectivity) of the main text. This shows that the regions that constitute the group-level maps are more likely to be statistically significant

in single-subject data than other cortical regions. However, these regions are significant only in 26 (skewness), 19 (kurtosis), and 18 (edge connectivity) subjects. This analysis thus shows that third- and fourth-order connectivity can be detected in single-subject data, but only in a minority of the subjects. Furthermore, in the above statistical analysis, no correction for multiple corrections was applied. Such a correction requires the approximation of extreme percentiles of the estimators' sampling distributions, namely $2.5/360 = 0.0069\%$ and $100 - 97.5/360 = 99.7292\%$, which requires an unfeasibly large number of bootstrap realizations. When the sampling distributions of the plug-in estimators are approximated by normal distributions, the statistical threshold is set to $\alpha = 0.01$, and a Bonferroni-correction is applied, we found no significant third- and fourth-order connectivity in any of the 94 subjects.

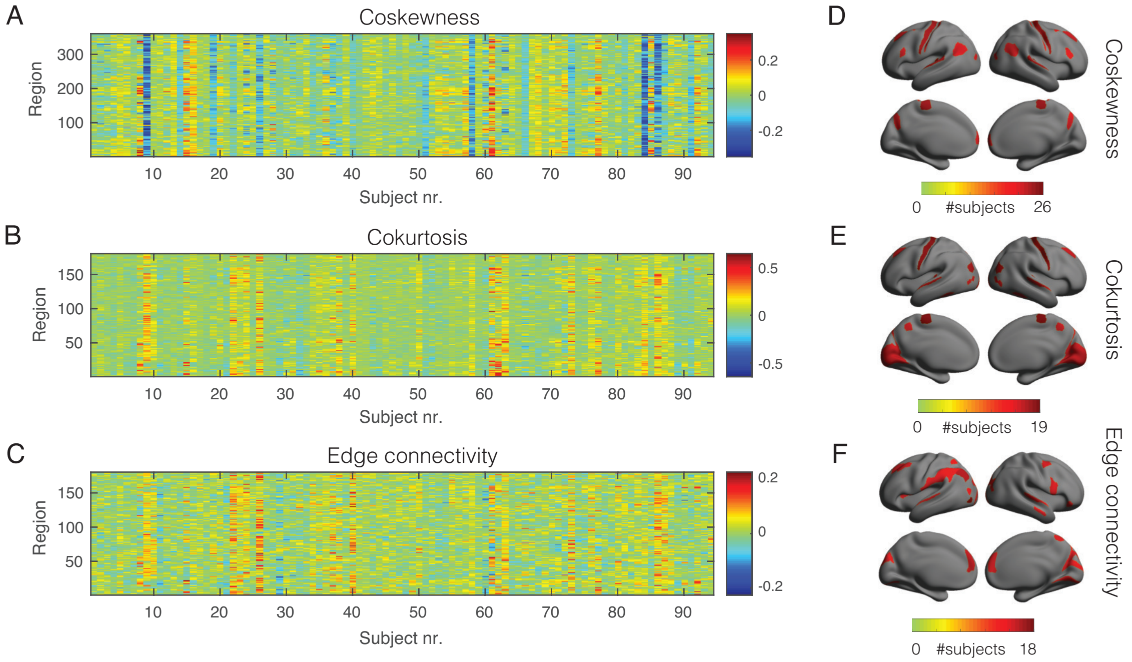

Figure 1: *Higher-order connectivity in single-subject data.* A. Coskewness maps for each of the 94 HCP subjects. B. Cokurtosis maps for each of the 94 HCP subjects. C. Edge connectivity maps for each of the 94 HCP subjects. D. Number of subjects (out of the 94 subjects) for which the coskewness with the frontal eye fields was significant. E. and F. Same format as D but for the cokurtosis and edge connectivity, respectively. All maps were seeded at the left and right frontal eye fields. For the coskewness maps, the target varied over all cortical regions, and for the cokurtosis and edge connectivity maps, the target varied over all homologous region-pairs. The significance threshold was set to $\alpha = 0.05$ (uncorrected).

To check if the results are substantially different when using different regions, we repeated the above

analysis using the precunei as seed regions, instead of the frontal eye-fields. The results were comparable with those for the frontal eye-fields. Specifically, the average spatial correlation between the maps from different subjects is 0.03 ± 0.23 (coskewnss), 0.03 ± 0.15 (cokurtosis), and 0.02 ± 0.13 (edge connectivity). We again computed the spatial correlations of the maps with those obtained from a separate scanning session. The subject-averaged spatial correlations were 0.14 ± 0.26 (coskewness), 0.06 ± 0.18 (cokurtosis), and 0.15 ± 0.15 (edge connectivity). For comparison, the subject-averaged spatial correlation between the second-order correlations maps obtained from two separate scanning sessions is 0.67 ± 0.12. Lastly, the maximum number of subjects for which regions were significant are 27 (skewness), 20 (kurtosis), and 18 (edge connectivity).

E. Symmetric random variables have vanishing odd multivariate moments

We show that the multivariate moments of odd order of three random variables with symmetric marginal distributions are zero. In particular, the coskewness of three symmetric random variables is zero. Let X, Y, and Z be zero-mean random variables with joint probability distribution $f(x, y, z)$ and suppose that their marginal distributions are even, i.e.

$$\int\int f(x,y,z)dxdy = \int\int f(x,y,-z)dxdy,$$

and the same for x and y and let

$$\langle XYZ\rangle = \int\int\int xyzf(x,y,z)dxdydz,$$

be the third moment of (x, y, z). This implies that

$$\int\int xyf(x,y,z)dxdy = \int\int xyf(x,y,-z)dxdy,$$

for all functions $g(x, y)$ and the same for x and y. Substituting $(x', y', z') = (x, y, z)$ shows that

$$\langle XYZ\rangle = \int\int\int(-x')(-y')(-z')f(x',y',z')dx'dy'dz'.$$

Note that this step uses the oddness of the moment. Subsequently applying Eq. (15) to Eq. (16) and using Eq. (12), i.e. for each of the three variables, shows that $\langle XYZ\rangle = -\langle XYZ\rangle$ and hence $\langle XYZ\rangle = 0$. The general case is proved in the same way.

F. Finite moments of resting-state fMRI signals

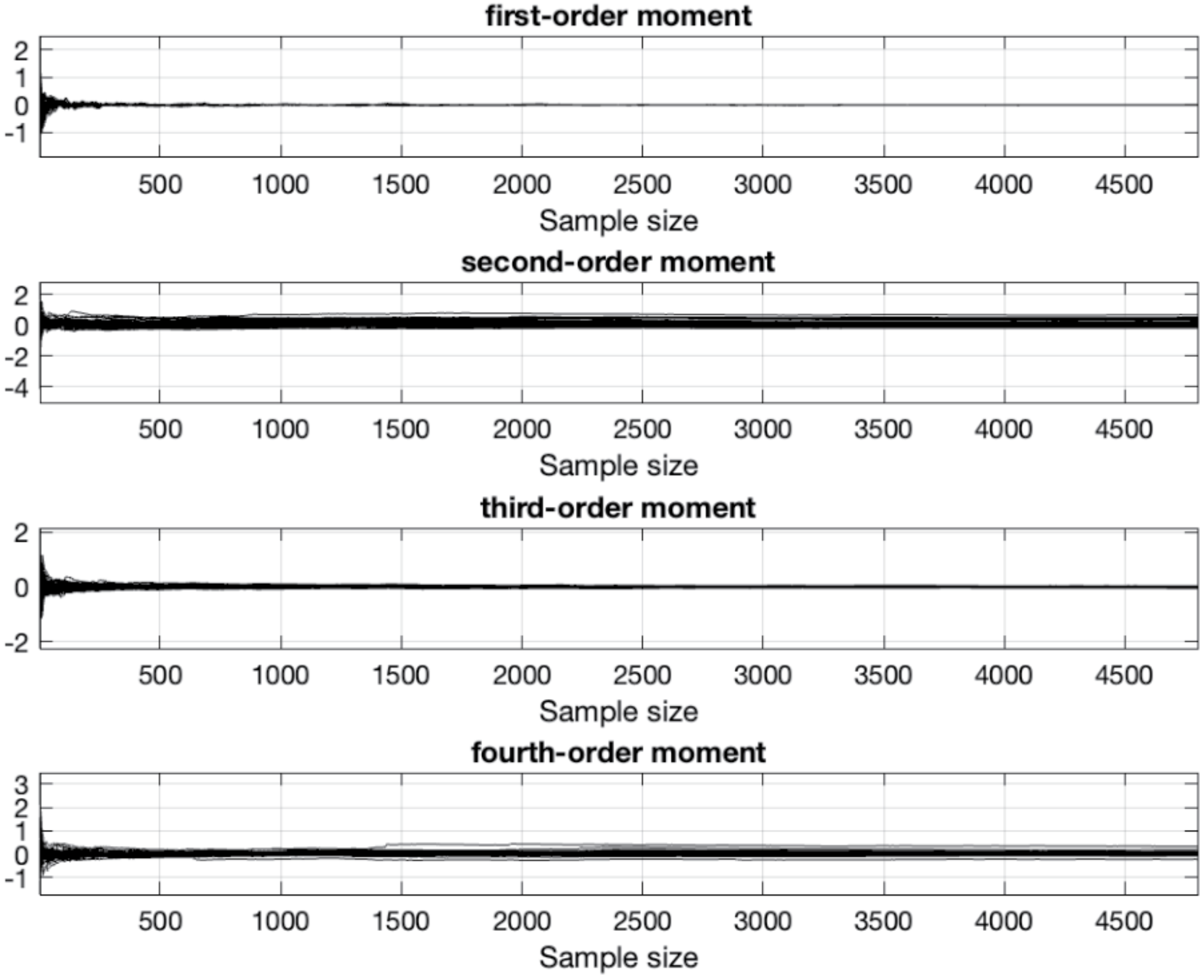

Figure 1: *Sample moments as a function of sample size.* Shown are the first sample moment (i.e. the sample mean), the second-order sample moment (i.e. the sample covariance), the third-order sample moment and the fourth-order sample moment, as functions of sample size, for 10^4 randomly selected quadruples of regions of a single HCP subject. The sample size ranges from 1 to 4800, which corresponds to four scanning sessions (each HCP subject has four scanning sessions of 1200 samples). Note that the sampling variability of the moments is large for small sample sizes, but stabilizes when the sample size becomes larger, indicating that the corresponding theoretical moments are finite.
